# Rician Likelihood Loss for Quantitative MRI With Self‐Supervised Deep Learning

**DOI:** 10.1002/nbm.70136

**Published:** 2025-09-03

**Authors:** Christopher S. Parker, Anna Schroder, Sean C. Epstein, James Cole, Daniel C. Alexander, Hui Zhang

**Affiliations:** ^1^ UCL Hawkes Institute, Department of Computer Science University College London London UK

**Keywords:** deep learning, diffusion MRI, intravoxel incoherent motion, likelihood, mean squared error, quantitative MRI, Rician, self‐supervised

## Abstract

Quantitative MR imaging with self‐supervised deep learning promises fast and robust parameter estimation without the need for training labels. However, previous studies have reported significant bias in self‐supervised parameter estimates as the signal‐to‐noise ratio (SNR) decreases. A possible source of this bias may be the choice of the mean squared error (MSE) loss function for network training, which is incompatible with MR magnitude signals. To address this, we introduce the Rician likelihood loss for self‐supervised learning, which explicitly accounts for the distribution of MR magnitude signals during training. We develop a stable and accurate numerical approximation of the negative log Rician (NLR) likelihood loss and compare its performance against the MSE loss using the intravoxel incoherent motion (IVIM) model as an exemplar. Parameter estimation performance was evaluated in simulated data and real data in terms of accuracy, precision and overall error by quantifying the bias, standard deviation and root mean squared error of network predictions against ground truth (or gold standard) values over a range of SNRs. Results show that self‐supervised networks trained with the NLR loss have increased accuracy (reduced bias) of IVIM diffusion coefficient at low SNR, at the cost of reduced precision. As SNR increases, the performance of the NLR and MSE losses converges, resulting in estimates with higher accuracy, higher precision and lower total error. The NLR loss has potential for broad application in quantitative MR imaging by enabling more accurate parameter estimation from noisy data. The NLR loss is available as a Python package: https://pypi.org/project/RicianLoss.

## Graphical abstract

We introduce a numerically accurate and stable negative log Rician (NLR) likelihood loss for quantitative MR imaging with self‐supervised deep learning. Self‐supervised neural networks trained with the NLR loss have reduced bias in intra‐voxel incoherent motion diffusion coefficient at low signal‐to‐noise ratio (SNR) compared to the traditional mean squared error loss; and at high SNR their performance converges. The NLR loss has potential for broad application in quantitative MR imaging by enabling more accurate parameter estimation from noisy data (https://pypi.org/project/RicianLoss).

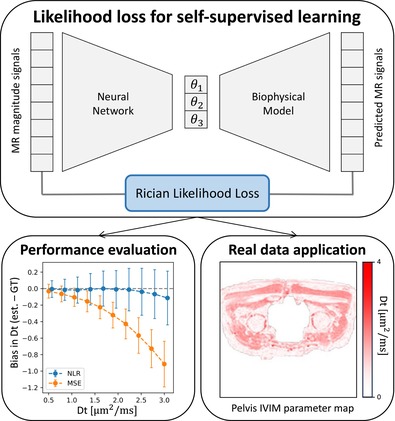

**Abbreviations**
IVIM: intravoxel incoherent motion
MLE: maximum likelihood estimation
MSE: mean squared error
NLR: negative log Rician
qMRI: quantitative MRI
SNR: signal‐to‐noise ratio

## 1 Introduction

Quantitative MRI (qMRI) aims to estimate and map tissue properties of interest using biophysical models that relate these properties, as parameters, to measured MR signals [1, 2]. Parameter estimation enables assessment of the spatial and subject‐wise variability in target tissue properties, which can inform understanding of disease processes and support objective, data‐driven evaluation of patients from MRI data. For reliable inference and interpretation of tissue properties with qMRI, accurate parameter estimation is essential.

Biophysical model parameters are traditionally estimated by optimising a fitting objective function for each voxel of an MR image. This can be time‐consuming, taking minutes to hours for a single volume. Furthermore, for complex non‐linear models with many parameters, the objective function is often nonconvex and contains local minima, increasing the propensity for estimation errors. Deep learning approaches have been developed to overcome some of these limitations, demonstrating orders of magnitude improvement in inference speed and more robust parameter estimates [3, 4, 5, 6, 7, 8, 9].

Deep learning approaches, which train neural networks to predict biophysical model parameters directly from the measured MR data, can be divided into two categories: supervised and self‐supervised. Supervised approaches aim to minimise some measure of loss between the parameter estimates and the parameter ground truth labels, or gold standard reference values, that accompany the MR data. Self‐supervised approaches instead aim to minimise a measure of loss between the measured MR data and the MR signals predicted by the parameter estimates. Self‐supervised approaches have the advantage over supervised approaches in that they do not require accompanying labels and can therefore be trained using only the MR data, making them applicable to a wider range of datasets. However, previous studies using self‐supervised deep learning for qMRI have reported increasing bias in parameter estimates as SNR decreases [4, 5, 7].

A significant source of bias may be the choice of loss function used for network training. So far, self‐supervised networks have been almost exclusively trained with the mean squared error (MSE) loss function [4, 5, 6, 7, 8, 9, 10, 11]. Minimising the MSE is equivalent to least squares estimation, which is unbiased only when the noise is centred on the true signal, that is, for Gaussian noise with zero mean [12, 13, 14]. However, qMRI models typically utilise magnitude signals, which become increasingly non‐Gaussian and shifted from the underlying noise‐free signal as SNR decreases [15, 16]. This violation of the least squares assumption may in part explain the increasing bias in parameter estimates as SNR decreases.

To mitigate this bias, we introduce the Rician likelihood loss for self‐supervised deep learning. The likelihood‐based loss transforms the aim of self‐supervised learning into reconstructing the noise‐free signal with the highest probability, as opposed to the input measures themselves. Training then becomes equivalent to maximum likelihood estimation (MLE), which is known to be unbiased under self‐consistent conditions (i.e., with the correct noise model) and with sufficient data [17, 18]. By explicitly accounting for the distribution of MR measures and its dependence on SNR, the proposed loss aims to mitigate bias associated with applying the MSE to non‐Gaussian MR signal measures.

We demonstrate the approach for magnitude data following a Rician distribution, although the method can be readily extended to other distributions. The Rician likelihood loss is applicable to magnitude data reconstructed using sum‐of‐squares reconstruction from a single complex image, the typical output from a single quadrature coil or SENSE reconstruction [19, 20, 21], both of which are commonly available acquisition techniques in clinical and research practice. Although previous studies have recognised the need for a Rician likelihood loss for training self‐supervised networks on MR magnitude images [22, 23], none have so far demonstrated a numerically stable and accurate implementation or analysed its potential benefit.

In this study, we describe the theoretical basis for the existence of bias when using the MSE loss on MR magnitude data that is Rician distributed and provide the rationale for its mitigation with the proposed approach. We then introduce a stable and accurate numerical approximation of the negative log Rician likelihood loss and empirically evaluate its performance using the intravoxel incoherent motion (IVIM) model as an exemplar qMRI application. Results show the loss mitigates bias associated with decreasing SNR and performs comparably to the MSE at high SNR.

## 2 Theory

### 2.1 Self‐Supervised qMRI

qMRI estimates the parameters of a biophysical model of the MR signal in an image voxel from a set of measured signals. The measured signals are acquired under varying experimental conditions to provide contrast for parameter estimation and are equal to or larger in number than the set of model parameters.

**FIGURE 1:**
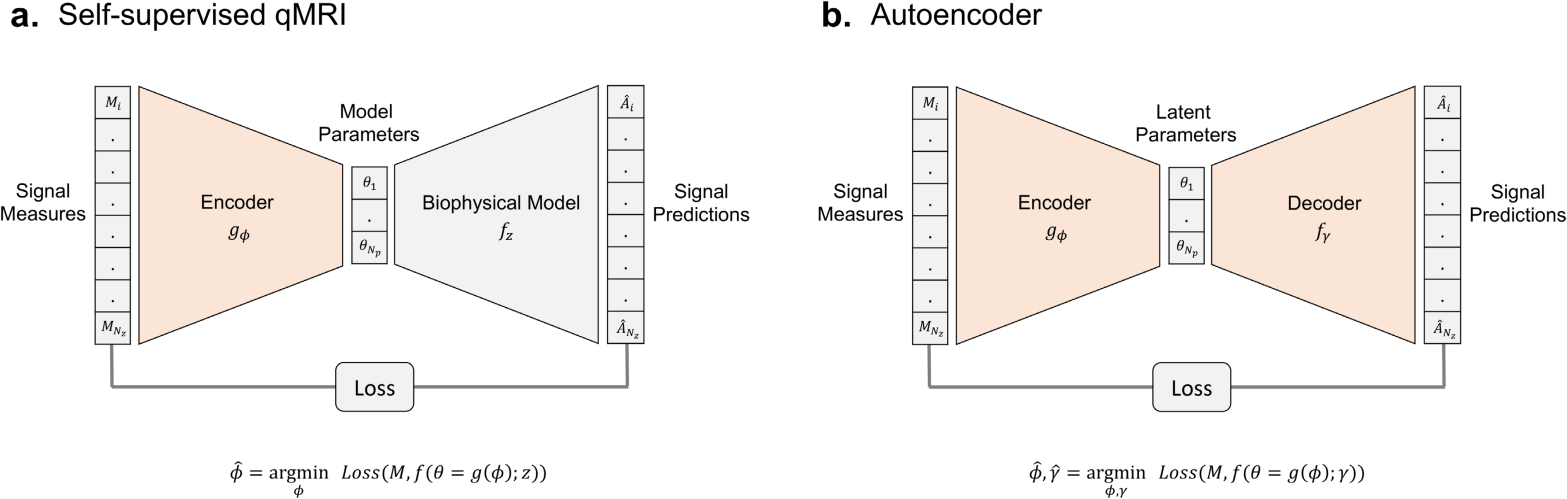
Comparison between self‐supervised qMRI and autoencoders in terms of network architecture and optimisation. The shaded orange region denotes the trained component of the architecture. (a) Self‐supervised qMRI. The encoder network parameters are trained to output the model parameters that minimise the loss between the signal measures and the signal predictions. Signal predictions are made under the model parameters and the corresponding biophysical model. (b) Autoencoders. The encoder and decoder network parameters are trained to minimise the loss between the signal measures and the signal predictions. Signal predictions are made under the encoded latent space parameters via the decoder.

In self‐supervised qMRI, a neural network is trained to predict parameters directly from a set of signal measures by minimising a measure of error between the signal predicted by the model parameters (corresponding to the predicted noise‐free signal) and the measured signals. This architecture mimics that of an autoencoder [24], with the input being the measured signals, the encoder being a fully connected neural network, the latent space being the model parameters, the decoder being the biophysical model and the output being the signal predictions (Figure 1).

### 2.2 MSE Loss Function

Self‐supervised neural networks are trained to minimise an amortised loss, which is the average of some measure of error between signal measures and signal predictions over a set of voxels. The MSE loss function, denoted $L_{\mathrm{MSE}}$, trains networks to minimise the MSE between the predicted and measured MR signals and is calculated as

(1) $$L_{\mathrm{MSE}}=\frac{1}{N}\sum_{j=1}^{N}\;\left[\;\frac{1}{N_{z}}\sum_{i=1}^{N_{z}}{\left(M_{i,j}-{\hat{A}}_{i,j}\right)}^{2}\right]$$

where M is the measured signal, $\hat{A}$ is the predicted signal, $N_{z}$ is the number of measured signals in a voxel and N is the number of voxels in the batch. The set of signal measures for a particular voxel j, denoted $\left\{M_{i,j}|\;i=1,\ldots ,N_{z}\right\}$, are acquired under varying experimental conditions $\left\{z_{i}|i=1,\ldots ,N_{z}\right\}$ and the set of corresponding signal predictions, $\left\{{\hat{A}}_{i,j}=f\left(\theta ;z_{i}\right)|i=1,\ldots ,N_{z}\right\}$, are generated from the chosen biophysical model with model parameters θ.

The global minimum of the MSE loss function occurs when the least squares estimate of the signal predictions is attained for each voxel. Least squares predictions closely match the measured data and are unbiased estimates of the noise‐free signal when the noisy measurement distribution is centred on the noise‐free signal (e.g., for signals corrupted by zero‐mean Gaussian noise). In other words, least squares estimators are unbiased when the mean of the noisy measures has an expected value equal to the noise‐free signal [12, 13, 14]. Least squares estimates are also maximum likelihood estimates under the condition of zero‐mean Gaussian noise [25]. However, in qMRI, magnitude signal measures are typically used, which are neither Gaussian nor centred on the noise‐free signal.

### 2.3 MR Magnitude Signals

**FIGURE 2:**
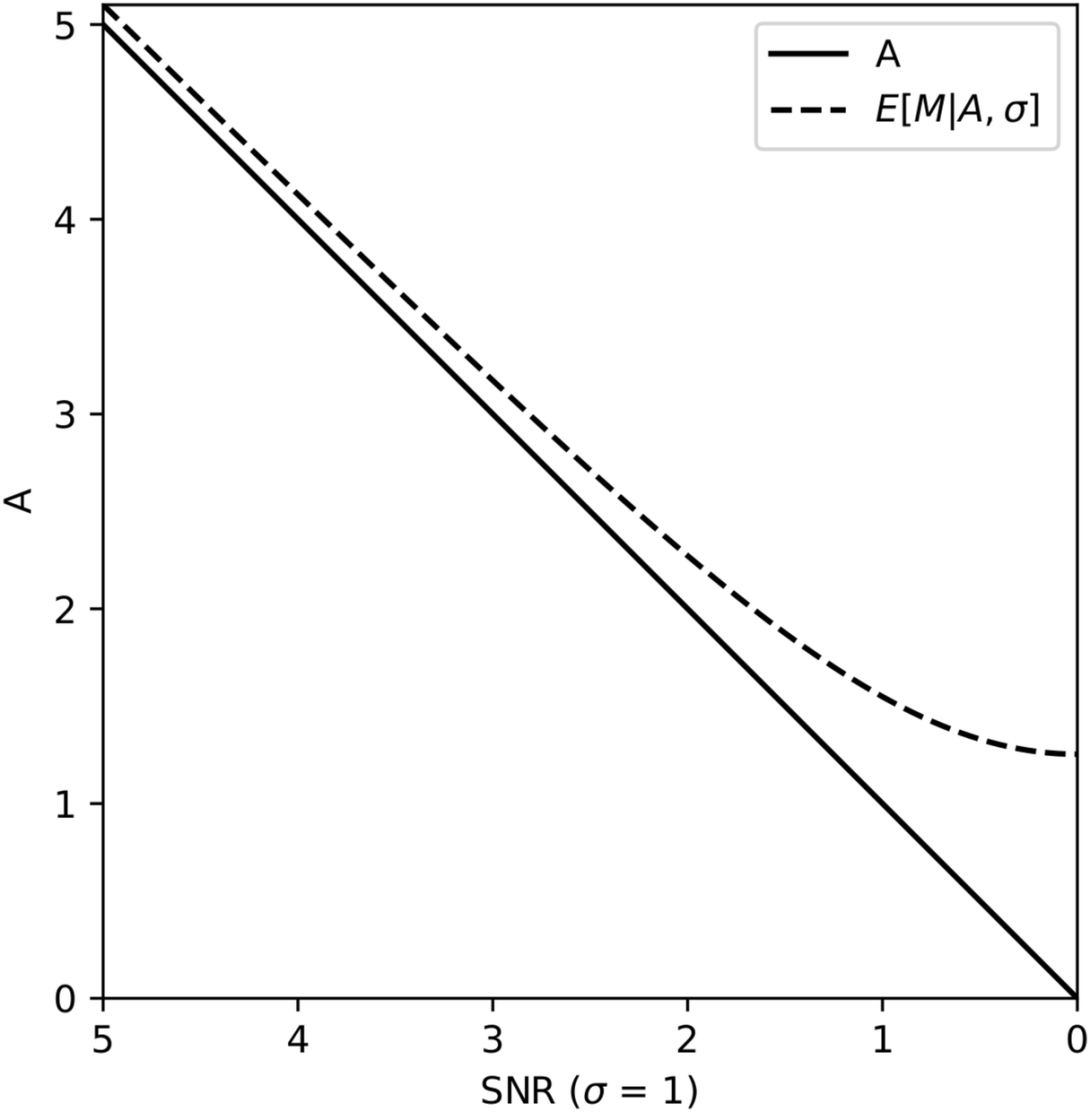
The expected value of the measured signal, $E\left[M|A,\sigma \right]$, as a function of SNR. $E\left[M|A,\sigma \right]$ corresponds to the centre of the noise and the least squares estimate of the noise‐free signal at a particular SNR. Its value increasingly overestimates A as SNR decreases.

The raw *k*‐space MR signal is represented as a complex number consisting of a real and imaginary component, each of which is corrupted by zero‐mean Gaussian noise and remains Gaussian following transformation to image space via the inverse Fourier transform [26]. Magnitude signals are typically reconstructed from the complex image space signals using the sum‐of‐squares reconstruction. When the MR data is acquired using a single quadrature receiver coil or reconstructed with a SENSE reconstruction, the magnitude of a single complex‐valued signal is taken at each voxel, $M=\sqrt{N_{R}^{2}+N_{I}^{2}}$, where $N_{R}$ and $N_{I}$ and are real Gaussian‐distributed random variables representing the real and imaginary components. In this case, the magnitude signal follows a Rician distribution [15] with probability density function:

(2) $$\begin{array}{c} p\left(M|A,\sigma \right)=\frac{M}{\sigma^{2}}e^{\frac{-\left(M^{2}+A^{2}\right)}{2\sigma^{2}}}I_{0}\left(\frac{\mathrm{MA}}{\sigma^{2}}\right) \end{array}$$

where $I_{0}$ is the modified Bessel function of the first kind with order zero, A is the true noise‐free signal that the biophysical model aims to predict and σ is the noise standard deviation. The centre of this distribution, $E\left[M|A,\sigma \right]$, corresponding to the expected least squares estimate of A, is always greater than A:

(3) $$\begin{array}{c} E\left[M|A,\sigma \right]=\sigma \sqrt{\frac{\pi }{2}}L_{\frac{1}{2}}\left(\frac{-A^{2}}{2\sigma^{2}}\right)>A \end{array}$$

where $L_{1/2}$ is the Laguerre polynomial. The lower the SNR, the greater the difference between A and the least squares estimate (Figure 2).

Figure 2 and Equation (3) show that biophysical models can achieve a lower MSE by predicting signals that are higher than the true noise‐free signal when the data is Rician distributed. In this case, the signal prediction and associated model parameter estimates are biased. Using the MSE loss for self‐supervised quantitative MR imaging is therefore likely to be a source of bias in parameter estimates when the SNR of the data is low.

### 2.4 Rician Likelihood Loss Function

An alternative method for parameter estimation is MLE. MLE aims to maximise the probability of the measured data under the predicted signal and noise. In layman's terms, the predicted signal determines the location and shape of the Rician distribution, and MLE places this predicted distribution over the observed magnitude signals. MLE is known to be statistically consistent, meaning that it is asymptotically unbiased when the model noise distribution matches that of the measured data [17, 18]. In other words, MLE estimates are unbiased under self‐consistent conditions with sufficient data. An equivalent justification of the asymptotic unbiasedness of MLE is that the distribution with maximum likelihood is as close as possible to the empirical data distribution [15]. By explicitly accounting for the distribution of measured MR signals, the likelihood‐based loss may mitigate bias associated with applying the MSE loss to non‐Gaussian distributed signals.

The Rician likelihood of a predicted signal is given by Equation (2). Assuming the noise components of the MR measures are independent of one another, the total likelihood across all signal predictions for a voxel is the product of likelihoods. Network training involves minimising a loss function; in this context, Rician likelihood may be maximised by defining the training loss as the negative of the Rician likelihood. The Rician likelihood, R, defined for a single voxel is

(4) $$\begin{array}{c} R=\prod_{i=1}^{N_{z}}p\left(M_{i}|{\hat{A}}_{i},\sigma \right) \end{array}$$

As a joint probability, Equation (4) is subject to numerical overflow because it requires taking the product of multiple probabilities. It is common, therefore, to instead minimise the negative of the logarithm of the Rician likelihood. The log transformation turns multiplication into summation, reducing its propensity to overflow. Being a positively monotonic function, the log transformation preserves the location of the loss's global minimum. The negative of the log Rician likelihood for a voxel is

(5) $$\begin{array}{c} -\log\left(R\right)=-\sum_{i=1}^{N_{z}}\log\left(\frac{M_{i,j}}{\sigma^{2}}\right)-\frac{\left(M_{i,j}^{2}+{\hat{A}}_{i,j}^{2}\right)}{2\sigma^{2}}+\log\left(I_{0}\left(\frac{M_{i,j}{\hat{A}}_{i,j}}{\sigma^{2}}\right)\right) \end{array}$$

### 2.5 Loss Function Specification—Practical Challenges

Implementing Equation (5) in practice is non‐trivial. A naïve implementation, which directly programmes the equation as specified, has been found to be numerically unstable [23]. Our own evaluation suggests this is because $\log\left(I_{0}\left(x\right)\right)$ and its gradient both contain modified Bessel functions, which grow exponentially with the input value.

To overcome this, several approaches that avoid the direct computation of $I_{0}\left(x\right)$ have been reported [22, 27, 28] (Table S1). We have found that none so far provide satisfactory performance in terms of both accuracy and stability across a wide range of SNRs (Figures S1 and S2 and Table S2). The approach based on log‐sum‐exp [22] has poor accuracy at high SNR; this may be improved, but at the cost of significantly longer computation time. Hankel's and related approximations [27, 28] have poor accuracy at low SNR. Furthermore, we have found that both are subject to overflow.

We propose a new approach that is numerically stable and highly accurate for a wide range of SNRs and can be readily implemented in common machine learning packages (Table S1, Proposed, Section 5.2). The approach utilises the exponentially scaled Bessel function $I_{0}^{e}\left(x\right)$ [29, 30], which approximates $I_{0}\left(x\right)$ as a weighted sum of Chebyshev polynomials, $T_{i}$, multiplied by $e^{x}$ (as introduced by Blair and Edwards [31] and Blair [32]):

(6) $$\begin{array}{c} \begin{array}{c} I_{0}^{e}\left(x\right)=e^{-x}I_{0}\left(x\right)≔\left\{\begin{array}{c} e^{-x}\;\left[e^{x}P_{L}\left(x\right)\right]=P_{L}\left(x\right)=\sum_{i=0}^{30}c_{i}^{L}\;T_{i}\left(x_{t}\right)\;0\leq x\leq 8\\ e^{-x}\;\left[e^{x}P_{H}\left(x\right)/\sqrt{x}\right]=P_{H}\left(x\right)/\sqrt{x}=\sum_{i=0}^{25}c_{i}^{H}\;T_{i}\left(x_{t}\right)/\sqrt{x}\;8<x<\infty \end{array}\right.\# \end{array} \end{array}$$

The coefficients are tailored for high accuracy over low and high input ranges. $x_{t}$ is x transformed to the range [−1,1] over which the Chebyshev polynomials are defined. The transformations and coefficients are given in (www.netlib.org/cephes) [29].

As shown in Equation (6), the exponentially scaled Bessel function is simply the $I_{0}\left(x\right)$ approximation with the exponential term removed. As $\log\left(I_{0}^{e}\left(x\right)\right)=\log\left(I_{0}\left(x\right)\right)-x$, then a numerically stable computation of the log Bessel that avoids direct computation of $I_{0}\left(x\right)$ is $\log\left(I_{0}\left(x\right)\right)=\log\left(I_{0}^{e}\left(x\right)\right)+x$. This formulation can be readily implemented in common machine learning packages, for example, in PyTorch using *torch.special.i0e*. The final implementation of the negative log Rician likelihood loss function (NLR) is

(7) $$\begin{array}{c} L_{\mathrm{NLR}}=\frac{1}{N}\sum_{j=1}^{N}\left[-\sum_{i=1}^{N_{z}}\log\left(\frac{M_{i,j}}{\sigma^{2}}\right)-\frac{M_{i,j}^{2}+{\hat{A}}_{i,j}^{2}}{2\sigma^{2}}+\log\left(I_{0}^{e}\left(\frac{M_{i,j}{\hat{A}}_{i,j}}{\sigma^{2}}\right)\right)+\frac{M_{i,j}{\hat{A}}_{i,j}}{\sigma^{2}}\right] \end{array}$$

$L_{\mathrm{NLR}}$'s global minimum is obtained when the MLE of the model parameters are predicted for each voxel. In this case, and under asymptotic conditions (as the number of measured data approaches infinity), the negative Rician loss function will train networks to predict unbiased parameter estimates. As it is not possible to assess the unbiasedness of $L_{\mathrm{NLR}}$ analytically for a finite number of measured signals per voxel, empirical observation using simulated data is required to assess estimation performance.

Note that the likelihood requires users to specify the value of the noise standard deviation, σ, which we assume to be constant over all voxels at a given SNR. Under this assumption, we use an unbiased method to estimate σ (see Section 3.3.3), although several other methods have also been proposed.

## 3 Methods

### 3.1 Overview

Performance of the NLR and MSE loss is evaluated and compared in synthetic and real data. Parameter estimation performance is compared in synthetic Rician‐distributed magnitude data in terms of accuracy, precision and total error with respect to the ground truth parameters. In real data, the two losses are applied to estimate qMRI parameters, and performance is evaluated in terms of precision across repeated scans. Performance is also compared against gold standard parameters derived from the super‐sampled dataset.

### 3.2 Quantitative MR Biophysical Model

We examine performance using the IVIM model [33, 34] as an exemplar qMRI model. The IVIM model is fitted to diffusion‐weighted imaging data, with the self‐supervised deep learning approach becoming an increasingly common fitting method [4, 5, 11].

The IVIM model accounts for two sources of water displacement in a voxel: (i) diffusion in tissue and (ii) perfusion in capillaries. Assuming isotropic Gaussian displacement in both cases, the diffusion‐weighted signal decays bi‐exponentially and is parameterised by the tissue diffusion coefficient $D_{t}$, capillary pseudo‐diffusion coefficient $D_{p}$, signal fraction of capillary pseudo‐diffusion f and the diffusion‐weighted signal with no diffusion weighting $S_{0}$. The predicted MR signal, $\hat{A}$, for the IVIM model is

(8) $$\begin{array}{c} \hat{A}=S_{0}\;\left(f\cdot e^{-b\;\left(D_{p}+D_{t}\right)}+\left(1-f\right)\cdot e^{-b\;D_{t}}\right) \end{array}$$

To estimate the IVIM parameters, multiple diffusion‐weighted signals are acquired across a range of *b*‐values. As the incoherent motion within capillaries due to perfusion is much faster than diffusion, $D_{p}$ is orders of magnitude larger than $D_{t}$, and relatively low *b*‐values are required to capture the rapid signal decay.

### 3.3 Self‐Supervised Neural Network

#### 3.3.1 Network Architecture

A feed‐forward deep neural network was constructed in PyTorch (v 1.21.1) to estimate voxel‐wise qMRI model parameters using self‐supervised learning. Following Barbieri et al. [4], the first demonstration of self‐supervised deep learning for qMRI, the network has one input layer, three fully connected hidden layers (the encoder) and one output layer (the model parameters).

The number of nodes in the input layer and in each hidden layer was set equal to the number of acquired MR signals per voxel. The number of nodes in the output layer of the encoder was set equal to the number of model parameters for the IVIM model, that is, four ($D_{t},D_{p},f$ and $S_{0}$). Encoder nodes used an exponential linear unit (ELU) activation function [35]. The predicted MR signal was generated from the output layer following the forward model (Equation 8). The *b*‐values for predicted signals were ordered identically to the *b*‐values for the input measurements. In order for the backpropagation to take approximately equally sized parameter update steps, *b*‐values were specified in units of ms/μm^2^ so that the diffusion coefficient model parameter $D_{t}$ and $S_{0}$ were of similar magnitude (0.4–3 μm^2^/ms and 0–1 a.u., respectively).

#### 3.3.2 Loss Functions

Networks were constructed with either the negative log Rician likelihood loss function, $L_{\mathrm{NLR}}$, given in Equation (7), or the MSE loss function, $L_{\mathrm{MSE}}$, given in Equation (1).

#### 3.3.3 Sigma Estimation

In our implementation, the NLR loss function requires a prior estimate of the noise standard deviation σ, assumed to be constant across voxels at a given SNR. In this case, σ may be estimated from a background image region where there is no signal. In such regions, the Rician distribution becomes a Rayleigh distribution, with expected mean:

(9) $$\begin{array}{c} E\left[M\right]=\sigma \sqrt{\frac{\pi }{2}} \end{array}$$

Using the sample mean estimate of $E\left[M\right]$, an estimate of σ is

(10) $$\begin{array}{c} \hat{\sigma }=\frac{\sum_{i=1}^{N_{\mathrm{BG}}}M_{i}}{N_{\mathrm{BG}}\sqrt{\frac{\pi }{2}}} \end{array}$$

where $N_{\mathrm{BG}}$ is the number of background voxels, which in this study was set to 10,000. Sijbers et al. [36] show that this estimate is unbiased when applied to Rayleigh‐distributed data. Other methods to estimate σ also exist, for example, Sijbers et al. and Rajan et al. [36, 37, 38].

### 3.4 Network Training

#### 3.4.1 Simulated MR Signals

Simulated MR signals were used for network training. At a given SNR (the SNR of a *b* = 0 ms/μm^2^ signal equal to 1, defined as SNR = 1/σ), measured MR signals were simulated for 200,000 training voxels. Ground truth model parameter values for the training data were sampled uniformly from 10 equidistant numbers covering the range of physiologically plausible values: for the IVIM model, [0.4, 3] μm^2^/ms for $D_{t}$, [10, 150] μm^2^/ms for $D_{p}$, [0.1, 0.5] for f and [0.8, 1.2] a.u. for $S_{0}$. For each voxel, diffusion MR signals were simulated under a range of *b*‐values and complex Gaussian noise was added with a standard deviation of σ before computing the magnitude to produce Rician‐distributed signal measures. For the IVIM model, 10 *b*‐values representative of typical clinical protocol [4, 11, 39] were chosen: 0, 10, 20, 30, 50, 80, 100, 200, 400 and 800 ms/μm^2^. Twenty noisy data instantiations (voxels) were simulated for each of the 10^4^ combinations of ground truth parameters. MR signal measures for an additional 1000 voxels were also simulated to calculate validation loss, which was used as termination criteria. Data were simulated for SNRs of 30, 20, 10, 7.5 and 5 by varying the σ used for data generation and self‐supervised neural networks were trained at each SNR. This range covers the SNRs commonly observed in real data (typically 10–30) and includes even lower SNRs to test the limits of parameter estimation performance. Note hereafter that the unqualified term ‘SNR’ will refer to the SNR of a signal = 1 at *b* = 0 ms/μm^2^, whereas ‘effective SNR’ will refer to the signal acquired at a particular *b*‐value and set of model parameter values.

#### 3.4.2 Optimisation

For each SNR, self‐supervised neural networks were trained on the set of simulated MR signals using either the NLR or MSE loss. Network weights and biases were updated using stochastic gradient descent via backpropagation. An Adam optimiser was used with the default PyTorch settings (learning rate = 0.001, betas = (0.9, 0.0999), weight decay = 0) [40]. Batch sizes of 256 voxels were used in each gradient descent step. To prevent dependence on the random initialisation of weights and biases, a common initialisation was used for both loss functions at each SNR. The common initialisation was the trained network with the lowest validation loss over 16 training repetitions with the NLR loss. Training was terminated if the validation loss did not improve for 50 consecutive epochs or if a total of 300 training epochs were reached.

### 3.5 Performance Evaluation in Synthetic Data

For each SNR, 200,000 unseen simulated test voxels were produced identically to the training data (uniformly sampled across 10 equidistant numbers within the specified parameter ranges) but with different noise instantiations. The network was trained at the corresponding SNR and then predicted the model parameter values from the test signals, and the error was calculated (estimate minus ground truth). Parameter estimation performance was evaluated in detail at one representative low and one high SNR and then examined across a range of SNRs.

Parameter estimation performance was evaluated at SNR = 10 (representing typical low SNR) and SNR = 30 (representing typical high SNR). Metrics of accuracy and precision were calculated as a function of the ground truth parameter value. For each unique parameter combination, bias (the average error) against ground truth was calculated to quantify accuracy (with lower values indicating higher accuracy), standard deviation was calculated to quantify precision (with lower values indicating higher precision) and root mean squared error (RMSE) was calculated to quantify overall error. To visualise these metrics and their variation for a particular parameter and ground truth parameter value, the average and standard deviation of each metric was calculated across the set of unique parameter value combinations (e.g., over the unique values of $S_{0},D_{p}$ and f for the case of visualising $D_{t}$ performance).

Performance was then studied across the full range of SNRs to observe the overall error trends. At each SNR, the distribution of all errors across all test voxels was plotted as a boxplot for SNRs of 30, 20, 10, 7.5 and 5. The accuracy and precision analysis utilise traditional model evaluation metrics, and the error boxplots provide additional information about the distribution. Reporting both allows comparison of findings to previous studies [4, 11].

### 3.6 Performance Evaluation in Real Data

Performance of the NLR and MSE loss was next assessed in real high SNR data consisting of a set of repeated IVIM diffusion‐weighted imaging acquisitions acquired in the pelvis of a healthy volunteer who gave their informed consent to be scanned.

The MR sequence, optimised for IVIM, was acquired on a wide‐boar 3T Phillips Ingenia scanner (Amsterdam, Netherlands) with *b*‐values of 0, 10, 20, 30, 50, 80, 100, 200, 400 and 800 μm^2^/ms, with five slices, 224 × 224 matrix, voxel size = 1.56 × 1.56 × 7 mm, TE = 76 ms, TR = 516 ms, scan time = 44 s, and was repeated 16 times in the same scan session (data available at https://github.com/seancepstein). A SENSE reconstruction with sum‐of‐squares was applied to produce magnitude images from the multiple complex image space data acquired in parallel, resulting in Rician distributed signals. This dataset uniquely allows us to quantify IVIM parameter estimation performance in terms of bias, precision and total error for signals expected to follow a Rician distribution.

All datasets were normalised by the signal in bladder water, such that water has a non–diffusion‐weighted signal of 1. Self‐supervised networks were then trained on synthetic data, as described in Section 3.3, with the NLR loss or MSE loss. A wider range of $S_{0}$ (0.1–1) was used when generating the training data in order to represent the wider range of non–diffusion‐weighted signals observed. Sigma was estimated in a background region‐of‐interest using Equation (10), yielding $\sigma =2.5\times 10^{-3}$ (SNR = 395), corresponding to high SNR. IVIM parameter estimation was then performed using the trained network and the normalised IVIM data for each scan repetition separately.

Parameter estimation performance in real data was assessed in three ways. Firstly, maps were visually compared against known pelvis anatomy. Secondly, the precision of parameter estimates was quantified over the 16 scan repetitions. Lastly, the accuracy (bias) and overall error (RMSE) with respect to the gold standard parameter values were also calculated for each voxel. The gold standard parameter values were derived by fitting the IVIM model to each voxel separately using MLE with the Rician likelihood using *scipy.optimise*. The Rician likelihood here used the same numerical approximation of the Bessel function as for self‐supervised training (Equation 6), but via *scipy.special.i0e* instead of *torch.special.i0e*, which are identical implementations of the Blair and Edwards approximation [31, 32] (www.netlib.org/cephes).

## 4 Results

### 4.1 Synthetic Data Experiments

#### 4.1.1 Training

The NLR loss had a similar training behaviour to the MSE loss in terms of convergence and training time. Both losses decreased rapidly during the first 50 epochs and converged slowly thereafter (Figure S3). Training typically terminated after reaching the maximum number of epochs, as opposed to the maximum patience threshold. Training time was 5.72 s/epoch for the NLR loss and 4.68 s/epoch for the MSE loss, corresponding to 28.6 and 23.4 min for 300 training epochs (post‐initialisation training), and 1 h 35 min and 1 h 25 m for both initialisation and training.

#### 4.1.2 Parameter Estimation Performance at Low SNR

**FIGURE 3:**
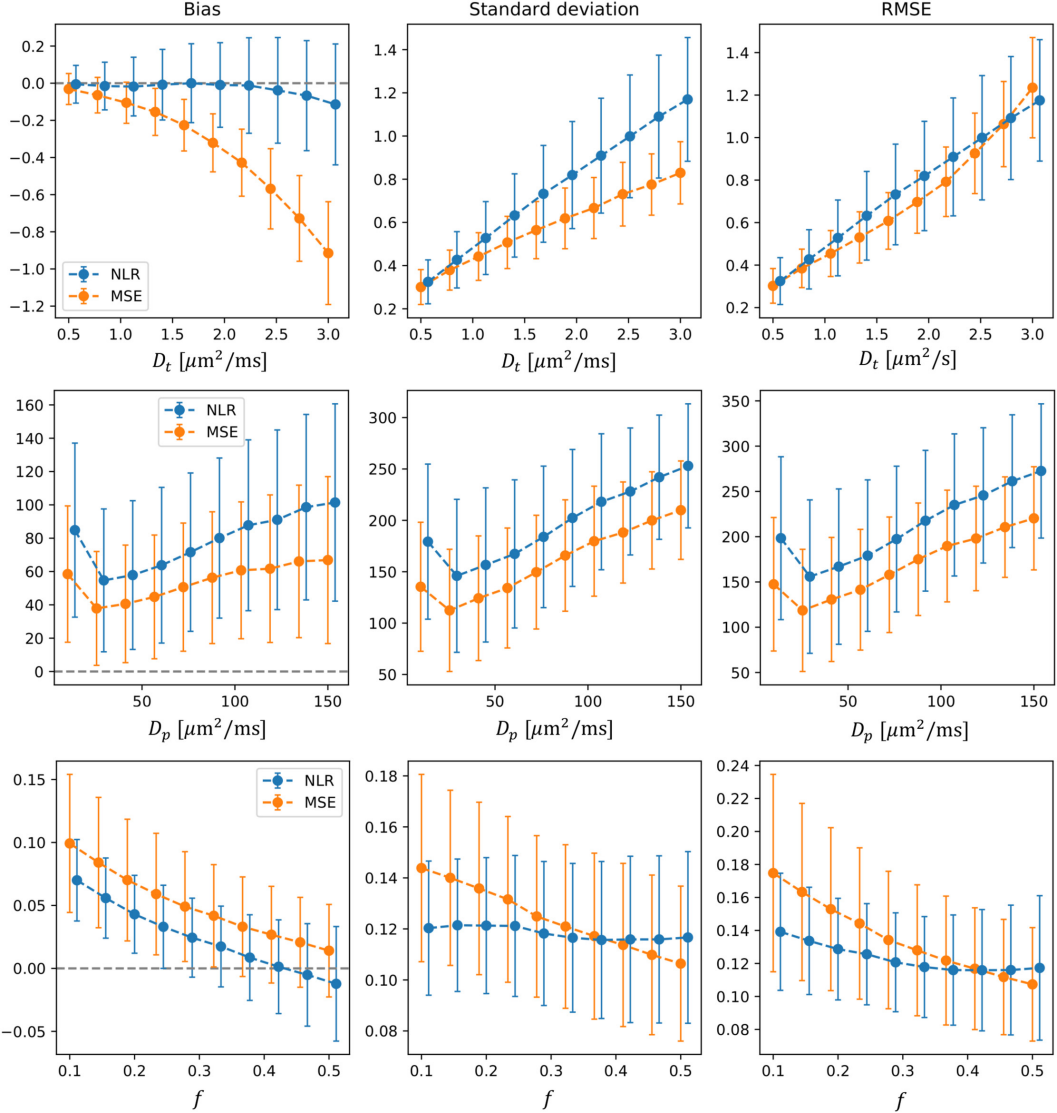
Comparison of estimation performance in synthetic data at low SNR (10) between self‐supervised networks trained with NLR and MSE loss for the IVIM model. Points and error bars show the mean and standard deviation of the performance metric across unique parameter combinations. NLR points and error bars have been jittered to the right to aid visualisation. *Y*‐axis units match those used for the *x*‐axis.

At a low SNR of 10, the MSE loss showed significant bias in IVIM diffusion coefficient that increased with higher ground truth values of the diffusion coefficient, that is, when the effective SNR was lower, with bias reaching an average of ~30% for $D_{t}$ (Figure 3). The NLR loss showed improved accuracy in $D_{t}$, reducing bias to a maximum average of ~3%. However, this reduction in bias coincided with a loss of precision (higher standard deviation).

The NLR showed marginally improved accuracy and precision over the MSE for the pseudo‐diffusion fraction f, whereas the MSE showed higher accuracy and precision for the pseudo‐diffusion coefficient $D_{p}$. For both losses, precision decreased and overall error increased with higher diffusion and pseudo‐diffusion coefficients, as seen from the increasing standard deviation and RMSE.

#### 4.1.3 Parameter Estimation Performance at High SNR

**FIGURE 4:**
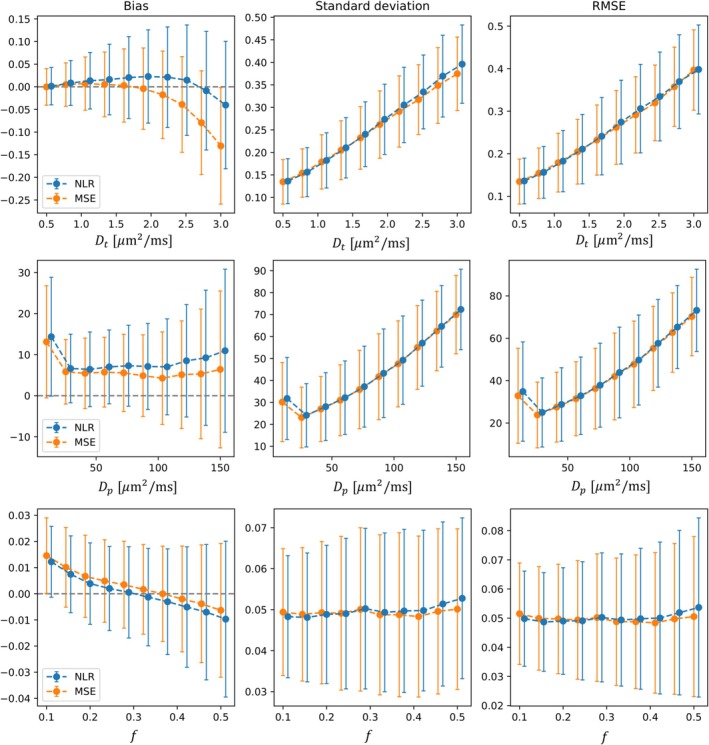
Comparison of estimation performance in synthetic data at high SNR (30) between self‐supervised networks trained with NLR and MSE loss for the IVIM model. Points and error bars show the mean and standard deviation of the performance metric across unique parameter combinations. NLR points and error bars have been jittered to the right to aid visualisation. *Y*‐axis units match those used for the *x*‐axis.

At a high SNR of 30, the NLR and MSE losses performed similarly, with both showing improved accuracy and precision compared with low SNR. However, even at an SNR of 30, the MSE loss showed a higher degree of systematic underestimation of $D_{t}$ as diffusivity increased (effective SNR decreased; Figure 4, upper left), with bias reaching a maximum of ~5% at high diffusivities. In comparison, the NLR loss showed no significant bias in IVIM diffusion coefficient estimation at high SNR for the range of $D_{t}$ investigated.

Accuracy of the other IVIM model parameters $D_{p}$ and f was approximately equal for MSE and NLR losses at high SNR. Precision and overall error were approximately equal between the losses and increased with higher values of the diffusion and pseudo‐diffusion coefficients (lower effective SNR).

#### 4.1.4 Error Distribution Across SNRs

**FIGURE 5:**
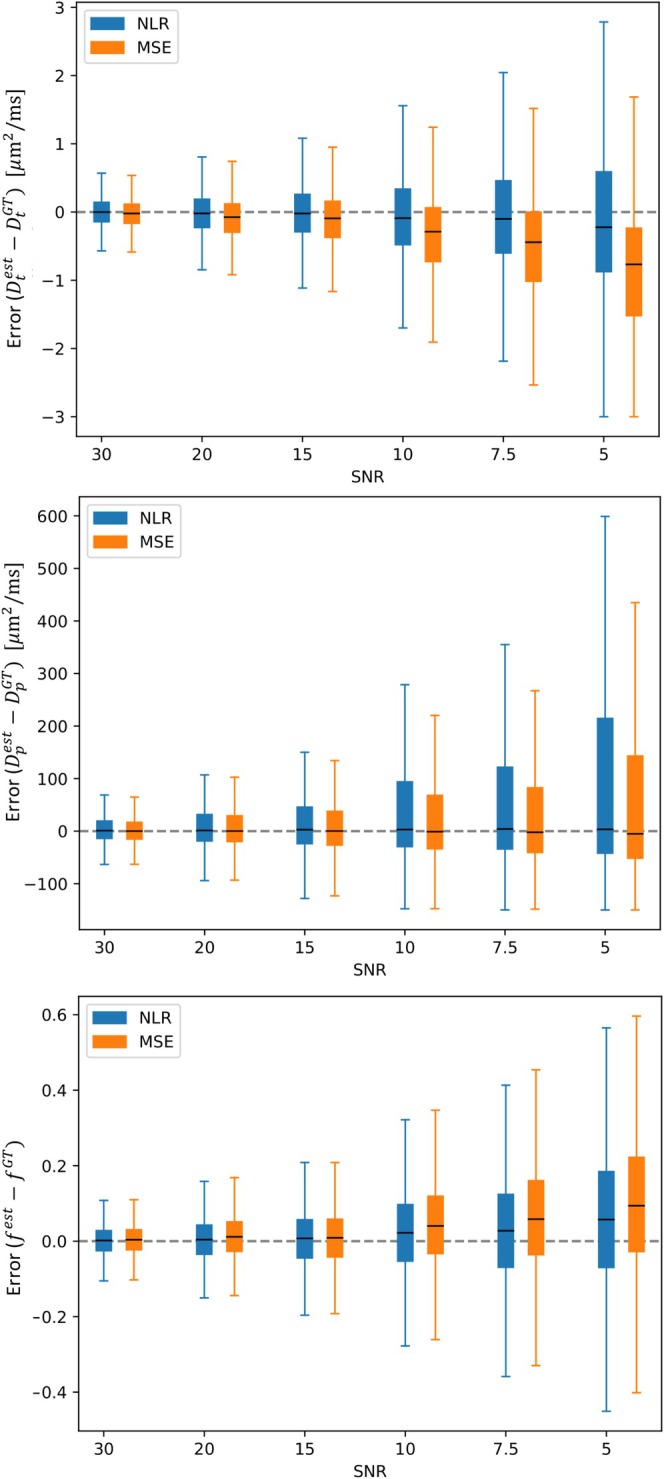
Boxplots of errors in parameter estimates for synthetic data at SNRs of 30, 20, 10, 7.5 and 5 for the IVIM model. The line shows the median error across all estimates and the box shows the interquartile range. Whiskers extend to the most extreme data point within 1.5 times the interquartile range from the median.

The summary of errors across SNRs (Figure 5) highlights similar accuracy trends to that observed at the representative low and high SNRs (Figures 3 and 4). Boxplots show that the MSE loss tended to increasingly underestimate $D_{t}$ as SNR decreased (median lower than zero, Figure 5), whereas the NLR was relatively more stable. A similar trend in accuracy is observed for f, with the median error of the NLR loss being closer to 0. Both losses perform similarly in terms of the median error for $D_{p}$.

As SNR decreased, both losses showed a reduction in precision (wider interquartile range) for all metrics, with the NLR loss showing marginally lower precision than the MSE loss for $D_{t}$ and $D_{p}$. Furthermore, both losses showed increasing overestimation of f as SNR decreased, but this overestimation was higher with the MSE than the NLR. As SNR approached 30, both losses converged to give comparable error distributions with approximately equal medians and interquartile ranges.

### 4.2 Real Data Experiments

**FIGURE 6:**
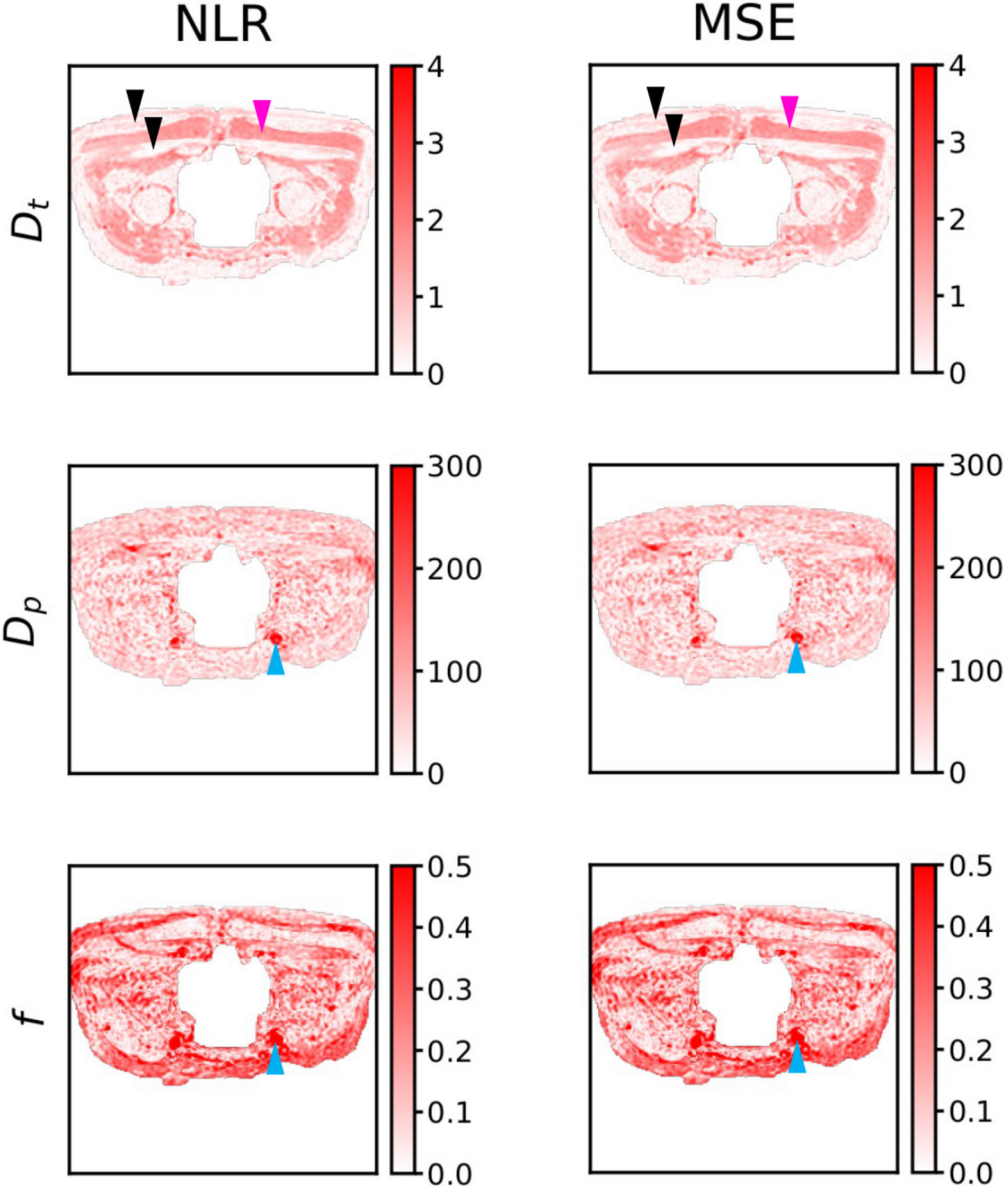
Example IVIM model predictions in real data for the first of 16 scan repetitions. MSE = mean squared error loss; NLR = negative log Rician loss. Arrows point to known pelvis anatomy (black to skin and fat, pink to bone, blue to veins/arteries). $D_{t}$ and $D_{p}$ colour bar units are μm^2^/ms.

Figure 6 shows IVIM parameter estimates for self‐supervised networks trained with the NLR or MSE loss for a single scan repetition of the real data acquisition. Both losses yield almost identical parameter estimates in this high SNR dataset. The spatial distribution of parameter estimates corresponds closely to known pelvis anatomy [9, 11]. For example, the apparent diffusion coefficient $D_{t}$ is lowest in fat and bone (black arrows) and highest in muscle (pink arrow), and the pseudo‐diffusion coefficient $D_{p}$ and the pseudo‐diffusion fraction f are highest in capillaries and veins (blue arrows).

**FIGURE 7:**
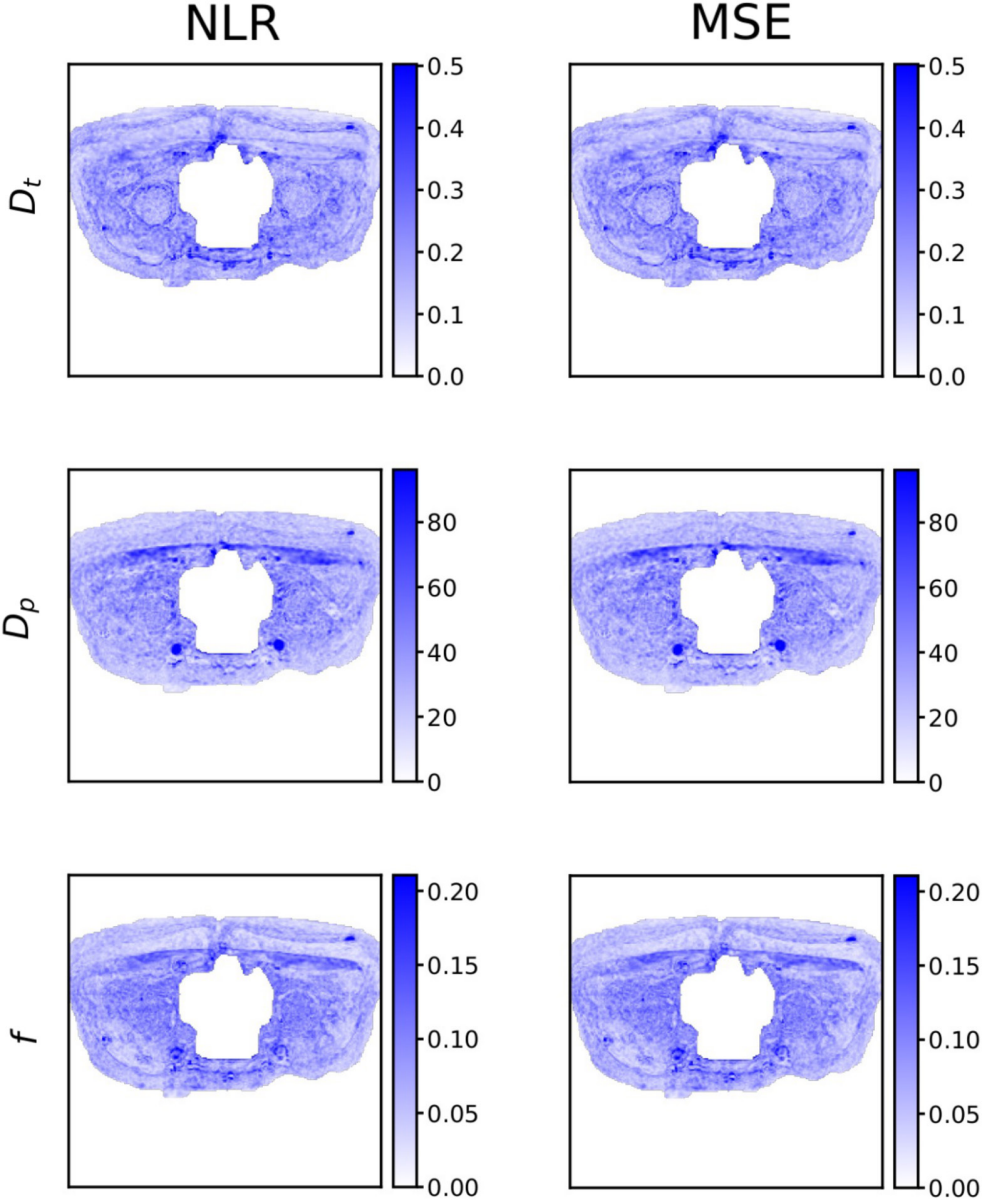
Maps showing the precision (standard deviation) of IVIM model parameter estimates obtained from self‐supervised deep learning in real data for self‐supervised networks trained with the NLR loss and MSE loss. $D_{t}$ and $D_{p}$ colour bar units are μm^2^/ms.

Figure 7 shows the precision of parameter estimates (standard deviation across the 16 repeat acquisitions) in real data. For both the NLR and MSE loss, the precision of all parameters is reasonably high. As in simulated data (Figures 3 and 4), precision tends to decrease for higher gold standard values of $D_{t}$ and $D_{p}$ (Figure S4).

For both losses, the parameter estimates also show high correlation to gold standard maps (Figures S5 and S6). Quantitative trends of bias and total error match closely to the high SNR synthetic data (Figure S4). For instance, higher $D_{t}$ is associated with a higher underestimation bias and higher total error.

## 5 Discussion

### 5.1 Summary

This study introduces the Rician likelihood loss for self‐supervised deep learning. The proposed loss aims to mitigate bias in quantitative MR imaging parameter estimates by accounting for the signal distribution and its dependence on SNR. Performance was evaluated and compared against the MSE loss for quantitative parameter estimation in the IVIM model. Results show the NLR loss mitigates bias in the IVIM diffusion coefficient at low SNR. At high SNR, both losses converge, resulting in similarly high performance, and in real data yield anatomically plausible parameter maps with high precision. Below, we discuss the numerical properties of the NLR loss, analyse the experimental results and highlight new opportunities and challenges that arise from this work.

### 5.2 Accuracy and Stability

We propose a stable and accurate implementation of the negative log Rician likelihood loss function for self‐supervised training. The implementation computes the log‐Bessel using the exponentially scaled Bessel function, resulting in high numerical stability, and approximates its value with Chebyshev polynomials, which are shown to be highly accurate (Figures S1 and S2). We observed high stability up to an SNR of 40 and high accuracy, with no quantitative difference from the ground truth, up to an SNR of 20, which was the maximum SNR at which the ground truth definition converged. These properties permit calculation of the loss over a wide range of SNRs, including those encountered in practical diffusion MR imaging scenarios. Previous implementations were found to be either inaccurate or numerically unstable for these SNR ranges. Recently, Simpson et al. [22] reported an approximate Rician log‐likelihood for self‐supervised learning based on finite series summation and applied this to image registration. However, as described in Section 2.5, this finite series implementation shows imperfect accuracy at high SNR and some numerical instability at low SNR (SNR < 5).

### 5.3 Synthetic Data Experiments

The NLR loss improved the accuracy of IVIM diffusion coefficient estimation compared with the MSE when SNR was low or the signal decayed more rapidly. Underestimation of the diffusion coefficient, corresponding to the overestimation of the signal, with the MSE loss in low SNR Rician‐distributed data, is consistent with findings from Epstein et al. [11], Barbieri et al. [4] and Zhou et al. [10]. The NLR loss mitigates this bias by accounting for the increasing non‐Gaussian and upward shift of the measured MR magnitude signals away from the noise‐free signal as the SNR decreases [15]. The MSE loss, on the other hand, minimises the residual error regardless of the data distribution, thereby underestimating the rate of signal decay and hence underestimating the diffusion coefficient. However, the gain in diffusion coefficient accuracy with the NLR loss is accompanied by a reduction in precision. A major source of this reduced precision is that the NLR loss enables a wider range of predictions by virtue of it avoiding the consistent underestimation of signal attenuation associated with the MSE loss. The apparent trade‐off between bias and variance, which we note is not directly analogous to the bias‐variance trade‐off observed in conventional model fitting [41], allows users to choose the loss function most suited to their study aims, for example, to maximise accuracy or precision of the IVIM diffusion coefficient.

Although MLE is asymptotically unbiased, some bias remained in parameter estimates with the NLR loss at low SNR. This is attributable to the finite number of MR signals acquired in a voxel: MLE applied to finite data samples is not guaranteed to yield unbiased estimates [42]. A potential strategy to address the residual bias may be to incorporate prior knowledge of the expected distribution of parameter values into the loss and maximise the posterior probability of estimates in a Bayesian framework. Our preliminary work suggests that it is feasible to incorporate the likelihood loss into variational autoencoders to predict the maximum a posteriori estimate and the parameter posterior distribution [43].

We observed that diffusivity parameter prediction performance on synthetic data tended to decrease at higher ground truth diffusivities for low SNR data for both losses (Figure 3), in agreement with trends reported in Epstein et al. [11]. At low SNR and high diffusivities, the effective SNR is low, making optimal signal predictions more susceptible to noise. $D_{p}$ estimation was relatively more biased and more variable than $D_{t}$, which is explained by two factors. Firstly, the error distributions for $D_{p}$ were heavily skewed towards over‐estimated values, increasing the bias and standard deviation metrics (Figure S7). Analysis of median errors shows that high $D_{p}$ bias at low SNR is at least partly attributable to this upwardly skewed error distribution (Figures S8 and S9). This skew likely arises from the long tail of the likelihood function at higher values than the ground truth (Figure S10). Secondly, the $D_{p}$ parameter has a relatively small effect on the signal compared with $D_{t}$: Larger relative changes in $D_{p}$ than in $D_{t}$ are needed to produce a given change in the signal prediction (Figure S11). The skewed error distribution and lower signal effect of $D_{p}$ make it particularly susceptible to high bias at low SNR.

Both loss functions demonstrate improved and convergent performance at high SNR. As SNR increases, the Rician distribution converges towards Gaussian and both the maximum likelihood and least squares estimates converge. This is because the noise‐free signal (the maximum likelihood estimate under self‐consistent and asymptotic conditions) becomes closer to the expected value of the measured data (the expected least squares estimate). However, even at a high SNR of 30 the MSE loss shows bias in parameter estimates when the effective SNR is low (Figure 4), in which case the NLR produces marginally more accurate estimates of $D_{t}$.

### 5.4 Real Data Experiments

We quantitatively evaluated the performance of the NLR and MSE loss in real‐world data using a super‐sampled IVIM dataset. In this dataset, both losses generated anatomically plausible maps with high correspondence to known pelvis anatomy, demonstrating the feasibility of applying the NLR loss in practice. The convergent performance of the losses as SNR increases was confirmed to extend beyond the highest SNR investigated in simulation experiments—in the super‐sampled dataset (SNR = 395), both losses gave near‐identical parameter estimates. The high SNR of the IVIM data was achieved using multi‐coil parallel imaging that optimised for higher SNR as opposed to reduced scan time. Furthermore, the NLR loss was numerically stable at this high SNR.

Precision of the NLR loss was reasonably high in real data, with a standard deviation of approximately < 20% of the gold standard reference value for $D_{t}$ and f, and < 50% for $D_{p}$ (Figures 7 and S4). However, this precision was lower than that observed in the high SNR simulated data. This could be due to a combination of factors. The training data for real data experiments had a wider range of $S_{0}$ than in the simulation experiments, resulting in a lower sampling density of the parameter space. In addition, some physiological features of tissue within the pelvis, such as the presence of anisotropic muscle fibres, connective tissue and larger more coherent vascular structures, are not accounted for by the IVIM model. Within‐scan temporal variation in IVIM tissue properties, such as an increase in capillary perfusion with body temperature [44], is another potential source of difference between the real data and the simulated training data.

Comparison of network estimates to the ground truth (gold standard in real data) revealed a similar pattern of bias between simulation and real data experiments. However, some regions showed higher bias in real data than expected, given the bias reported for the high SNR simulations. One reason for this is the lower density of training data for the real data experiments. Another explanation could be the reliability of the gold standard parameters themselves, which are subject to estimation errors due to the dependence of the conventional voxel‐wise MLE fitting on the correct estimation of the noise model and its hyperparameters (see Section 5.5). In fact, the issue of how to obtain gold standard parameters for real datasets is an active research area [45], and in addition to conventional fitting to super‐sampled data, alternative approaches are also available (e.g., comparison to histology data, or evaluation in phantom specimens). Despite this, the real data experiments confirmed the convergent behaviour of the two losses and the numerical stability of the NLR loss at high SNR.

Constructing the training datasets for the real data application required estimation of both sigma and the range of $S_{0}$. In our work, we estimated sigma following Equation (10) and the range of $S_{0}$ from the spatial variation of the mean of the unattenuated b = 0 volumes. Estimation of $S_{0}$ using this method is expected to be robust in high SNR data, where the mean is equal to the noise‐free signal. However, in lower SNR data, the mean is no longer equal to the noise‐free signal and alternative methods may be more suitable. Furthermore, in real data, we expect that sigma estimation, which in our method assumes a high degree of background homogeneity, may be more challenging than $S_{0}$ estimation (Section 5.5.2).

### 5.5 Challenges and Opportunities

#### 5.5.1 Data Distribution

The NLR loss is designed for Rician‐distributed MR magnitude data. This distribution is expected to arise for magnitude signals derived with sum‐of‐squares [20] reconstruction from a single complex image. This is often the case for MR data acquired with a single quadrature coil, reconstructed from multiple coils using SENSE [19], or following adaptive combined reconstruction of GRAPPA‐acquired data [46], all of which are common acquisition scenarios in clinical and research practice. However, oftentimes the data may not be Rician distributed.

Non‐Rician data may arise from either the reconstruction process or post‐reconstruction image preprocessing. For GRAPPA reconstructions [47], the magnitude data are expected to follow a noncentral chi distribution [16, 21]. The two distributions are not necessarily the same—the Rician distribution is a special case of the noncentral chi distribution with two degrees of freedom (corresponding to a single complex image). Therefore, the Rician likelihood loss may not always be applicable to GRAPPA reconstructed magnitude data. In this case, the loss may be adapted to a noncentral chi likelihood. Our preliminary report investigates this possibility [48] and demonstrates the feasibility of extending the likelihood loss concept to other data distributions. The preliminary work demonstrates that assuming a Rician distribution when the data is in fact noncentral chi distributed can increase bias, suggesting that the best performance for likelihood‐based losses is achieved when using the correct noise model.

Preprocessing steps, such as artefact reduction or image alignment, may also alter the data distribution. However, because of the complexity within and heterogeneity between different preprocessing algorithms, the shape of the final distribution is rarely known. Interpolation and averaging are common preprocessing steps, which, due to the central limit theorem, are likely to result in Gaussian‐distributed data. Yet, many preprocessing algorithms attempt to retain data non‐Gaussianity, for example, by ensuring signal nonnegativity. Determination of noise characteristics following preprocessing, and subsequent design of appropriate likelihood functions for those cases, will increase the range of datasets in which likelihood‐based deep learning losses can be applied.

#### 5.5.2 Sigma Estimation

In our current implementation, the NLR loss assumes that sigma (the standard deviation of the Gaussian component) is constant across voxels and estimates its value prior to training. We observed that estimating sigma from background voxels was highly accurate in simulated data (data not shown). However, in real data sigma estimation is likely to be more challenging due to factors such as limited field of view and the presence of background artefacts like ghosting. With a limited field of view (i.e., fewer background voxels), the estimated sigma is more prone to mis‐estimation. We explored this by evaluating NLR performance with varying sigma mis‐estimation (Figures S12 and S13). With increasing sigma under‐estimation, NLR loss performance converged to match the MSE loss. When sigma was overestimated, diffusivity parameters became increasingly overestimated. At high SNR, sigma mis‐estimation had less effect. We conclude that correct sigma estimation is most important at low SNR. Beyond mis‐estimation, background artefacts such as ghosting could make a priori sigma estimation unusable. Strategies to estimate sigma solely from anatomical signals during training, for example, by adapting the network architecture to learn sigma and model parameters simultaneously, may be useful in this case. A number of alternative noise characterisation methods are also available that report robust sigma estimation in real data [49, 50].

We emphasise that in order to simplify the loss formulation and training strategy, our approach assumes a constant sigma in the training and testing datasets. This is a limitation of our approach: Previous studies have shown that, as a result of post‐acquisition image preprocessing, in practice sigma commonly varies spatially across the imaged volume [49]. This scenario motivates the future development of algorithms that learn spatially varying noise hyperparameters and incorporate these into the training and inference.

#### 5.5.3 Conventional Fitting

In addition to self‐supervised learning, the NLR can also be used for conventional voxel‐wise fitting (i.e., Equation 4) of qMRI models. We demonstrate the use of the NLR loss for voxel‐wise IVIM fitting and compare performance against the MSE (Figures S14–S16). Results show that conventional fitting achieves similar performance to self‐supervised learning. As in the self‐supervised case, the NLR loss tended to show lower bias than MSE at low SNR, but with reduced precision. Interestingly, in conventional fitting at low SNR, $D_{p}$ tends to have lower bias for both losses than with self‐supervised learning, whereas at high SNR, the opposite was observed: Self‐supervised networks perform better. Self‐supervised networks have the advantage in terms of speed: Network‐based inference took only 0.00037 s/voxel, which is ~40 times faster than conventional fitting at 0.014 s/voxel. The NLR loss is of course applicable to other qMRI models beyond IVIM and has already been incorporated into conventional NODDI fitting [51, 52].

#### 5.5.4 Applications

The likelihood loss is naturally extendable to other data distributions and qMRI models. We evaluated the performance of the NLR loss using the IVIM model as an exemplar; the evaluation of performance in other biophysical models will be useful to determine the best use cases. The loss is expected to provide the most benefit for biophysical models that require highly attenuated low SNR signals. For example, diffusion MRI models of brain tissue that estimate the fibre distribution use high *b*‐values to generate contrast relating to axon orientation [53], or diffusion kurtosis imaging, which uses high *b*‐values to probe non‐Gaussian diffusion [54]. The loss may also be useful for quantitative T2 mapping, where long echo times are acquired to model compartments with long T2 [55]. Synthetic data experiments suggest the NLR provides the most benefit over the MSE in low SNR data. Future work will compare the performance of the two losses across different qMRI models and in real low SNR datasets.

Finally, the likelihood loss is not only applicable to qMRI but also to other self‐supervised deep learning tasks. The loss is as valid for the noise‐free signal predictions as it is for the underlying parameter estimates. Hence, the predicted signal likelihood can be maximised in a self‐supervised manner (e.g., with an autoencoder architecture; Figure 1) for the purpose of MR de‐noising. Furthermore, the loss is compatible with any generative model whose parameters are output from a neural network, making it well suited for self‐supervised deep learning of other latent structures from MR data.

## 6 Conclusion

This work introduces an accurate and stable negative log Rician likelihood loss for qMRI with self‐supervised deep learning. The NLR loss improves estimation accuracy of the IVIM diffusion coefficient in synthetic data experiments and generates plausible and precise parameter maps when applied to real data. The NLR loss has the potential to enable faster and more reliable quantitative analysis in noisy MR datasets, with broad application both within qMRI and beyond.

## Author Contributions

C.S.P.: conceptualisation, methodology, software, validation, formal analysis, investigation, writing – original draft, writing – review and editing, visualisation. A.S.: software, investigation. S.C.E.: data curation. J.C.: investigation. D.C.A.: funding acquisition. H.Z.: supervision.

## Conflicts of Interest

The authors declare no conflicts of interest.

## Supporting information


**Data S1:** Supplementary information.

**Table S1:** Approximate modified Bessel functions of the first kind with order zero and their logarithms. $T_{i}\left(x_{t}\right)$ are Chebyshev polynomials of the first kind evaluated at x transformed to the range $\left[-1,1\right]$ and $c_{i}^{*}$ are vectors of coefficients for the low or high ranges of x.
**Table S2:** Range of training data SNRs over which the loss functions incorporating the $\log\left(I_{0}\left(x\right)\right)$ approximations were numerically stable. Networks were trained on data with SNRs of (0, 40) in increments of 2.5. Training was stable if the loss function did not underflow or overflow. Proposed and Hankel‐2 were stable across the entire SNR range, whereas Series and Hankel‐1 were unstable at low SNR.

**Figure S1:** $\log\left(I_{0}\left(x\right)\right)$ approximations, as a function of input, x, and SNR $\approx \sqrt{x}$. In the left column, plots are a function of *x*. In the right column, plots are a function of SNR. The top and bottom rows show different ranges of SNRs—low and high, respectively. The ground truth (GT, blue line) was computed using 1000 summations of the series expansion, which converges in value for the range of x shown. Note the GT line is only partially visible as it overlaps with the approximations. In the bottom left plot (high SNR as a function of x), it is fully obscured by the Proposed and Hankel‐1 lines.

**Figure S2:** $\log\left(I_{0}\left(x\right)\right)$ approximations minus ground truth, as a function of input, x, and SNR $\approx \sqrt{x}$. In the left column, plots are a function of *x*. In the right column, plots are a function of SNR. The top and bottom rows show different ranges of SNRs—low and high, respectively. The ground truth was computed using 1000 summations of the series expansion, which converges in value for the range of x shown.

**Figure S3:** Training curves for the NLR and MSE loss on low SNR data. The upper panel shows loss curves with no initialisation, and the lower panel shows loss curves with initialisation.

**Figure S4:** Comparison of estimation performance in high SNR real data with respect to the gold standard maps between self‐supervised networks trained with NLR and MSE loss for the IVIM model. Points and error bars show the mean and standard deviation of the performance metric across binned parameter values. MSE points and error bars have been jittered to the right to aid visualisation.

**Figure S5:** Maps of parameter estimation performance (Bias, RMSE) in high SNR real data with respect to the gold standard (GS) parameter estimates for self‐supervised networks trained with the NLR loss. $D_{t}$ and $D_{p}$ are in units of μm^2^/ms.

**Figure S6:** Maps of parameter estimation performance (Bias, RMSE) in high SNR real data with respect to the gold standard (GS) parameter estimates for self‐supervised networks trained with the MSE loss. $D_{t}$ and $D_{p}$ are in units of μm^2^/ms.

**Figure S7:** Histograms showing the distribution of predicted parameters for networks trained with the NLR loss on low SNR (10) simulated data. Network predictions are shown for 100 noisy data instantiations for three different sets of ground truth parameter values (values indicated at the top of each row). The upper and middle panels show predictions for high and low $D_{t}$, and the lower panel shows predictions for low $D_{p}$. $D_{p}$ errors are highly skewed in all scenarios, leading to a higher bias (mean error) than median error (cf. Figures 3, 4 S15 and S16). $D_{t}$ errors are less skewed and tend to decrease with lower diffusivities (lower $D_{t}$ or lower $D_{p}$).

**Figure S8:** Comparison of estimation performance in synthetic data at low SNR (10) between self‐supervised networks trained with NLR and MSE loss for the IVIM model, in terms of median and interquartile range. Points and error bars show the mean and standard deviation of the median or interquartile range across unique parameter combinations. NLR points and error bars have been jittered to the right to aid visualisation.

**Figure S9:** Comparison of estimation performance in synthetic data at high SNR (30) between self‐supervised networks trained with NLR and MSE loss for the IVIM model, in terms of median and interquartile range. Points and error bars show the mean and standard deviation of the median or interquartile range across unique parameter combinations. NLR points and error bars have been jittered to the right to aid visualisation.

**Figure S10:** Skewness of the likelihood function for $D_{t}$ and $D_{p}$ parameters. At greater values of $D_{t}$ and $D_{p}$ than the ground truth, the gradient of the likelihood function becomes smaller than at lower values than the ground truth. The signal was generated with ground truth parameters of $D_{t}=1.5\;\mu m^{2}/\mathrm{ms}$, $D_{p}=150\;\mu m^{2}/\mathrm{ms}$, f=0.2, $S_{0}=1$. Here, the *y*‐axis shows the negative log Rician likelihood (NLR loss).

**Figure S11:** Effect on the signal of varying $D_{t}$ and $D_{p}$. Lines show signal predictions for varying $D_{t}$ (left) and $D_{p}$ (right) values with nonvarying ground truth parameters of $D_{t}=1.0\;\mu m^{2}/\mathrm{ms}$, $D_{p}=50\;\mu m^{2}/\mathrm{ms}$, f=0.2, $S_{0}=1$. The wider range of signals observed for $D_{t}$ demonstrates that varying $D_{t}$ has a larger influence on the signal compared with $D_{p}$. *b*‐values are in units of ms/μm^2^.

**Figure S12:** Effect of sigma‐misestimation on performance of the NLR loss at low SNR (10). Sigma is misestimated by a factor of a half to a factor of two. Points and error bars show the mean and standard deviation of the performance metric across unique parameter combinations.

**Figure S13:** Effect of sigma‐misestimation on performance of the NLR loss at high SNR (30). Sigma is misestimated by a factor of a half to a factor of two. Points and error bars show the mean and standard deviation of the performance metric across unique parameter combinations.

**Figure S14:** Comparison of estimation performance in synthetic low SNR (10) data between conventional voxel‐wise fitting with NLR and MSE loss for the IVIM model. Points and error bars show the mean and standard deviation of the performance metric across unique parameter combinations.

**Figure S15:** Comparison of estimation performance in synthetic high SNR (30) data between conventional voxel‐wise fitting with NLR and MSE loss for the IVIM model. Points and error bars show the mean and standard deviation of the performance metric across unique parameter combinations.

**Figure S16:** Boxplots of fitting errors in parameter estimates from conventional voxel‐wise fitting for synthetic data at SNRs of 30, 20, 10, 7.5 and 5 for the IVIM model. The line shows the median error across all estimates and the box shows the interquartile range. Whiskers extend to the most extreme data point within 1.5 times the interquartile range from the median.

## Data Availability

The negative log Rician likelihood loss is available as part of the RicianLoss python package: https://pypi.org/project/RicianLoss, and includes example code and implementations in other machine learning libraries. The super‐sampled IVIM data is available at https://github.com/seancepstein.
