# Supplementary material for: Rician Likelihood Loss for Quantitative MRI With Self‐Supervised Deep Learning
Source: NMR Biomed. 2025 Sep 3;38(10):e70136. doi: 10.1002/nbm.70136 (PMC12421220; doi:10.1002/nbm.70136)
Supplement: Supplementary file 1 — Data S1: Supplementary information. [file NBM-38-e70136-s013.docx]

**Tables**

|  | $\boldsymbol{I}_{\boldsymbol{0}}\boldsymbol{(x)}$ | $\mathbf{log(}\boldsymbol{I}_{\boldsymbol{0}}\left( \boldsymbol{x} \right)\boldsymbol{)}$ |
| --- | --- | --- |
| Series (Simpson, 2020) | $\sum_{k=0}^{50} \frac{1}{4}\frac{\left( x^{2} \right)^{k}}{\left( k! \right)^{2}}$ | $\log\left( \sum_{k=0}^{50} \begin{aligned} exp(-k\log\left( 4 \right)+2k\log\left( x \right) \\ -2log \Gamma(k+1)) \end{aligned} \right)$ |
| Hankel-1 (Abramowitz & Stegun 1972) | $\frac{e^{x}}{\sqrt{2\pi x}}$ | $x-\frac{\log\left( 2\pi x \right)}{2}$ |
| Hankel-2^24^ (Andersson, 2008) | $e^{x}$ | $x$ |
| Proposed  (Blair, 1974) | $e^{x}\sum_{i=0}^{30} c_{i}^{(l)}T_{i}\left( x_{t} \right), x=[0,8]$  $\frac{e^{x}}{\sqrt{x}}\sum_{i=0}^{25} c_{i}^{(h)}T_{i}\left( x_{t} \right), x=(8,\infty)$ | $x+\log\left( \sum_{i=0}^{30} c_{i}^{\left( l \right)}T_{i}\left( x_{t} \right) \right), x=[0,8]$  $x+\log\left( \sum_{i=0}^{25} c_{i}^{\left( l \right)}T_{i}\left( x_{t} \right) \right)-\frac{\log\left( x \right)}{2}, x=(8,\infty)$ |

**Table S1.** Approximate modified Bessel functions of the first kind with order zero and their logarithms. $T_{i}\left( x_{t} \right)$ are Chebyshev polynomials of the first kind evaluated at $x$ transformed to the range $[-1,1]$ and $c_{i}^{*}$ are vectors of coefficients for the low or high ranges of $x$.

|  | **Stable training SNRs** |
| --- | --- |
| Series | [5, 40] |
| Hankel-1 | [10, 40] |
| Hankel-2 | (0, 40] |
| Proposed | (0,40] |

**Table S2.** Range of training data SNRs over which the loss functions incorporating the $\log(I_{0}\left( x \right))$ approximations were numerically stable. Networks were trained on data with SNRs of (0, 40] in increments of 2.5. Training was stable if the loss function did not underflow or overflow. Proposed and Hankel-2 were stable across the entire SNR range, whereas Series and Hankel-1 were unstable at low SNR.
