# Supplementary material for: Rician Likelihood Loss for Quantitative MRI With Self‐Supervised Deep Learning
Source: NMR Biomed. 2025 Sep 3;38(10):e70136. doi: 10.1002/nbm.70136 (PMC12421220; doi:10.1002/nbm.70136)
Supplement: Supplementary file 2 — Table S1: Approximate modified Bessel functions of the first kind with order zero and their logarithms. Tixt are Chebyshev polynomials of the first kind evaluated at x transformed to the range −1,1 and ci* are vectors of coefficients for the low or high ranges of x. Table S2: Range of training data SNRs over which the loss functions incorporating the logI0x approximations were numerically stable. Networks were trained on data with SNRs of (0, 40) in increments of 2.5. Training was stable if the loss function did not underflow or overflow. Proposed and Hankel‐2 were stable across the entire SNR range, whereas Series and Hankel‐1 were unstable at low SNR. [file NBM-38-e70136-s004.docx]

**Supplementary Material**

|  | $\boldsymbol{I}_{\boldsymbol{0}}\boldsymbol{(x)}$ | $\mathbf{log(}\boldsymbol{I}_{\boldsymbol{0}}\left( \boldsymbol{x} \right)\boldsymbol{)}$ |
| --- | --- | --- |
| Series (Simpson, 2020) | $\sum_{k=0}^{50} \frac{1}{4}\frac{\left( x^{2} \right)^{k}}{\left( k! \right)^{2}}$ | $\log\left( \sum_{k=0}^{50} \begin{aligned} exp(-k\log\left( 4 \right)+2k\log\left( x \right) \\ -2log \Gamma(k+1)) \end{aligned} \right)$ |
| Hankel-1 (Abramowitz & Stegun 1972) | $\frac{e^{x}}{\sqrt{2\pi x}}$ | $x-\frac{\log\left( 2\pi x \right)}{2}$ |
| Hankel-2^24^ (Andersson, 2008) | $e^{x}$ | $x$ |
| Proposed  (Blair, 1974) | $e^{x}\sum_{i=0}^{30} c_{i}^{(l)}T_{i}\left( x_{t} \right), x=[0,8]$  $\frac{e^{x}}{\sqrt{x}}\sum_{i=0}^{25} c_{i}^{(h)}T_{i}\left( x_{t} \right), x=(8,\infty)$ | $x+\log\left( \sum_{i=0}^{30} c_{i}^{\left( l \right)}T_{i}\left( x_{t} \right) \right), x=[0,8]$  $x+\log\left( \sum_{i=0}^{25} c_{i}^{\left( l \right)}T_{i}\left( x_{t} \right) \right)-\frac{\log\left( x \right)}{2}, x=(8,\infty)$ |

**Table S1.** Approximate modified Bessel functions of the first kind with order zero and their logarithms. $T_{i}\left( x_{t} \right)$ are Chebyshev polynomials of the first kind evaluated at $x$ transformed to the range $[-1,1]$ and $c_{i}^{*}$ are vectors of coefficients for the low or high ranges of $x$.


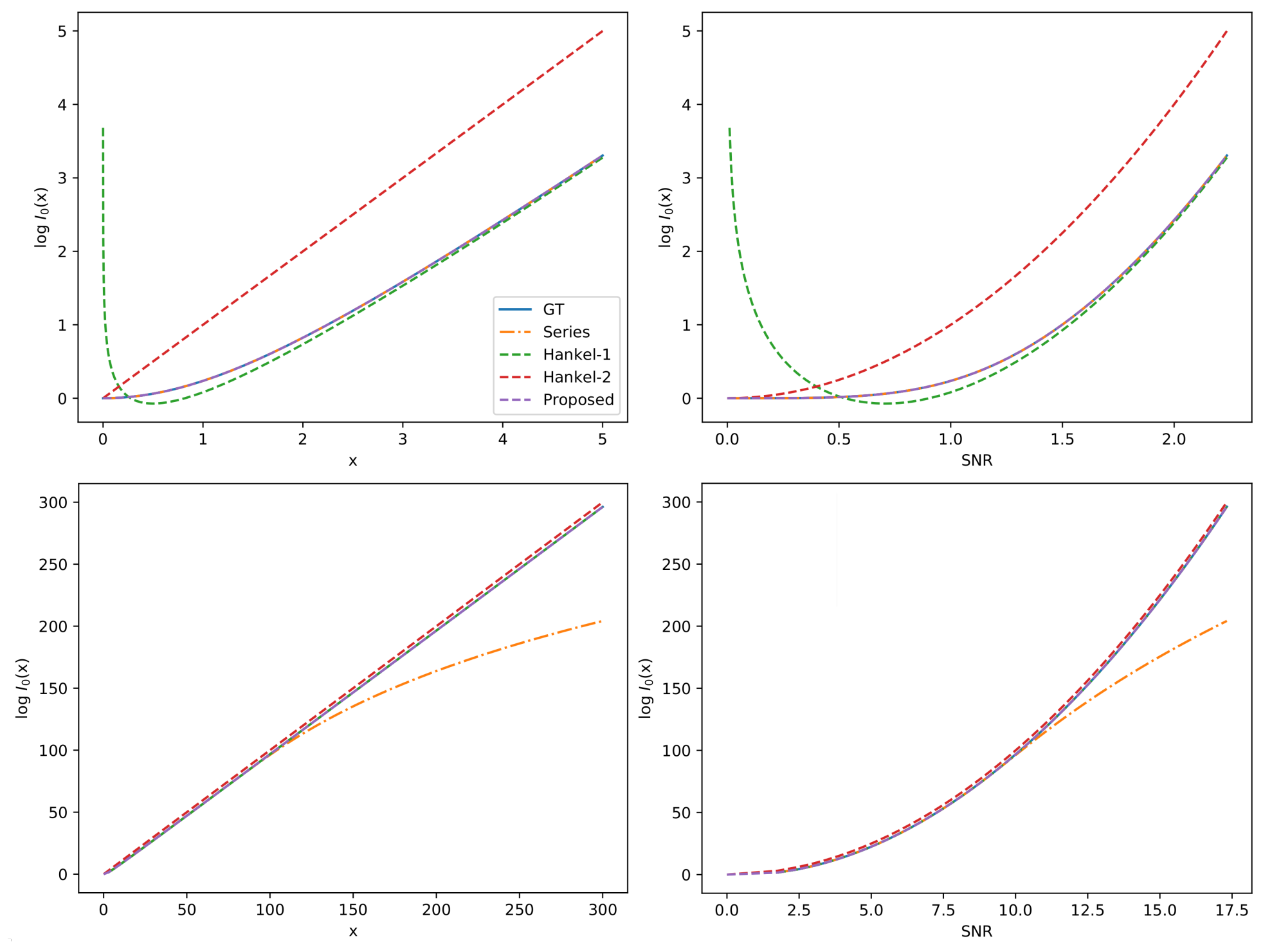


**Figure S1.** $\log(I_{0}\left( x \right))$ approximations, as a function of input, $x$, and SNR $\approx\sqrt{x}$. In the left column, plots are a function of x. In the right column, plots are a function of SNR. The top and bottom rows show different ranges of SNRs - low and high, respectively. The ground truth (GT, blue line) was computed using 1000 summations of the series expansion, which converges in value for the range of $x$ shown. Note the GT line is only partially visible as it overlaps with the approximations. In the bottom left plot (high SNR as a function of $x$), it is fully obscured by the Proposed and Hankel-1 lines.

**Figure S2.** $\log(I_{0}\left( x \right))$ approximations minus ground truth, as a function of input, $x$, and SNR $\approx\sqrt{x}$. In the left column, plots are a function of x. In the right column, plots are a function of SNR. The top and bottom rows show different ranges of SNRs - low and high, respectively. The ground truth was computed using 1000 summations of the series expansion, which converges in value for the range of $x$ shown.

|  | **Stable training SNRs** |
| --- | --- |
| Series | [5, 40] |
| Hankel-1 | [10, 40] |
| Hankel-2 | (0, 40] |
| Proposed | (0,40] |

**Table S2.** Range of training data SNRs over which the loss functions incorporating the $\log(I_{0}\left( x \right))$ approximations were numerically stable. Networks were trained on data with SNRs of (0, 40] in increments of 2.5. Training was stable if the loss function did not underflow or overflow. Proposed and Hankel-2 were stable across the entire SNR range, whereas Series and Hankel-1 were unstable at low SNR.

**Fig. S3.** Training curves for the NLR and MSE loss on low SNR data. Upper panel shows loss curves with no initialisation and lower panel shows loss curves with initialisation.


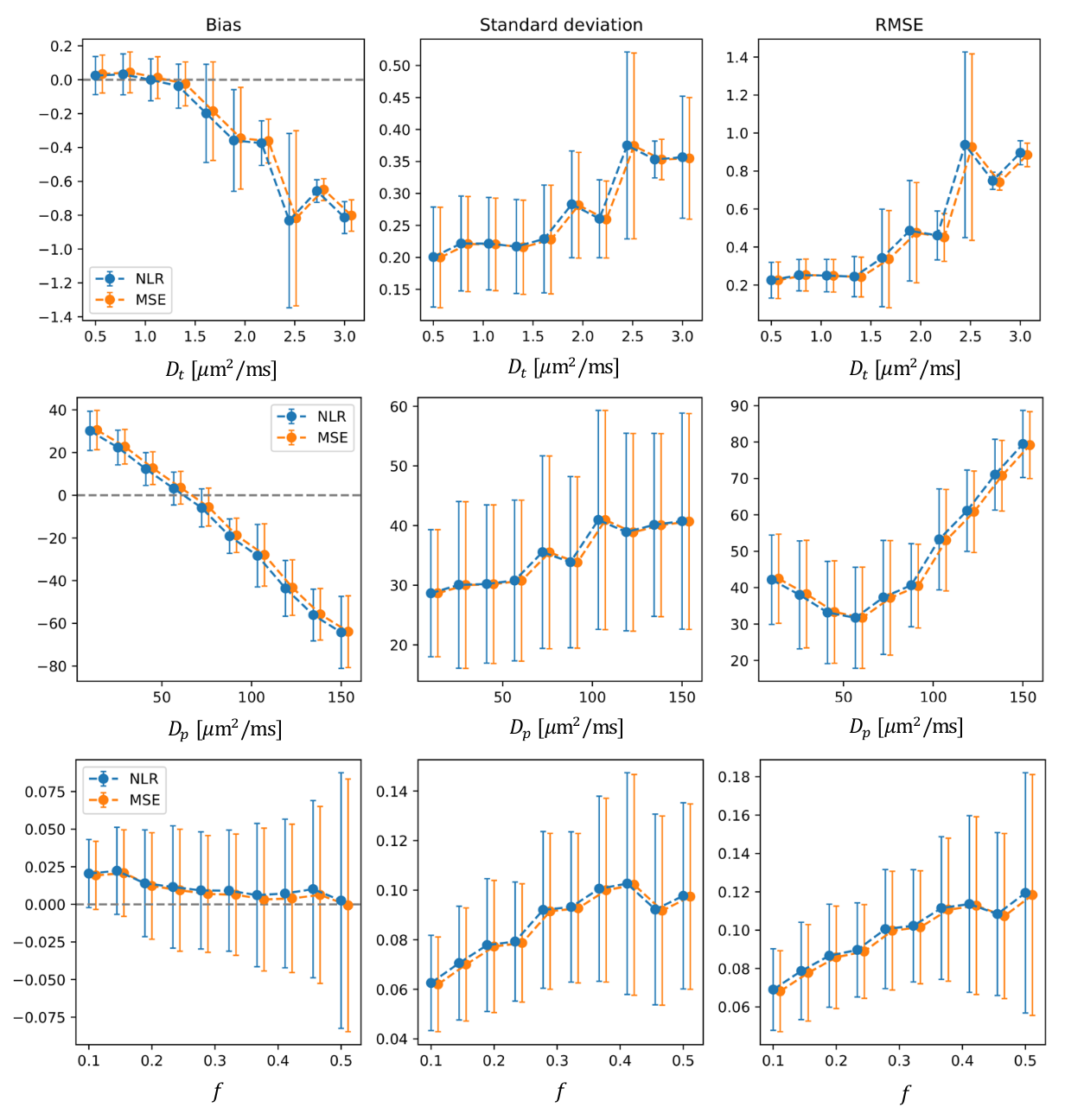


**Fig. S4.** Comparison of estimation performance in high SNR real data with respect to the gold standard maps between self-supervised networks trained with NLR and MSE loss for the IVIM model. Points and error bars show the mean and standard deviation of the performance metric across binned parameter values. MSE points and error bars have been jittered to the right to aid visualisation.


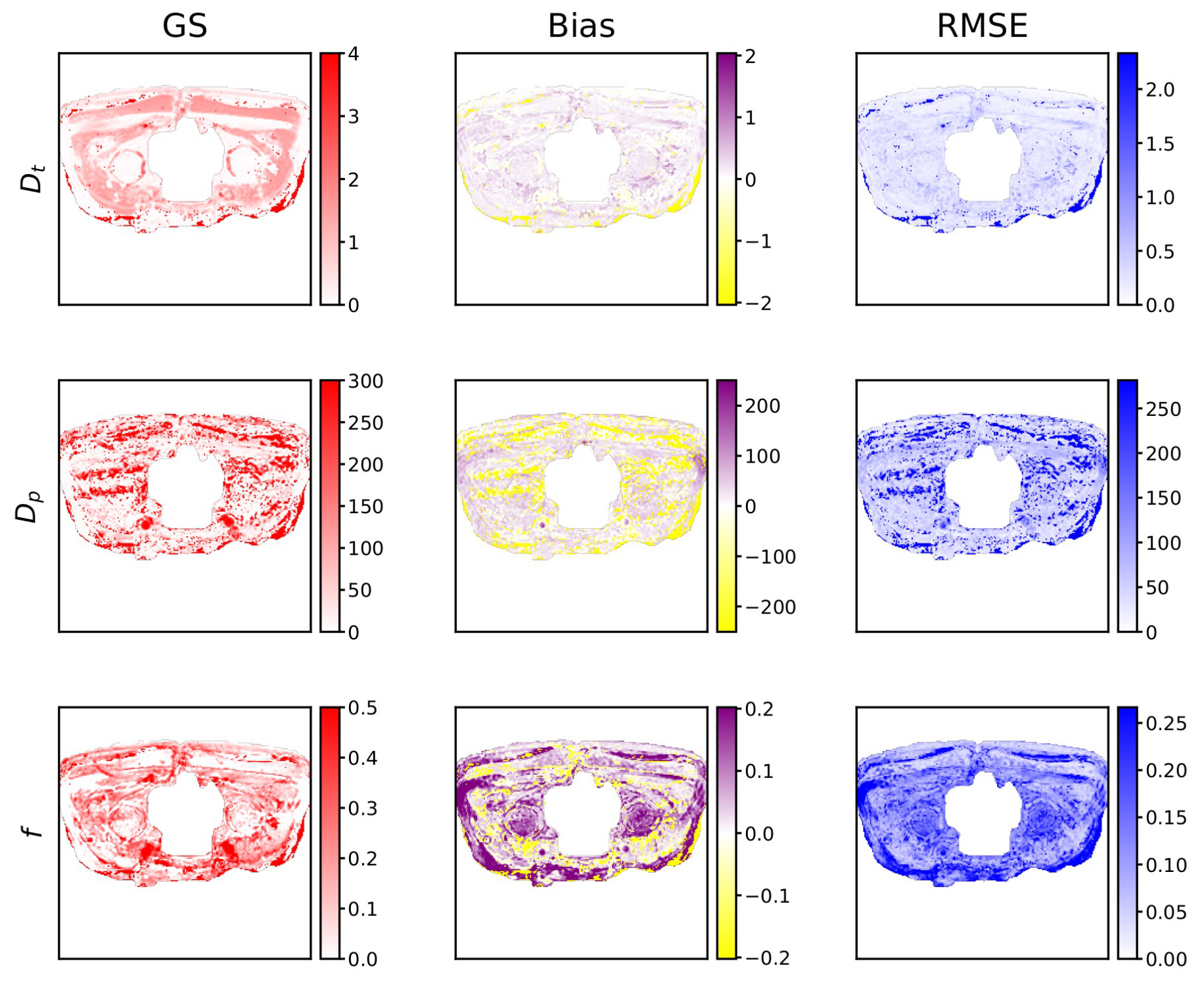


**Fig. S5.** Maps of parameter estimation performance (Bias, RMSE) in high SNR real data with respect to the gold standard (GS) parameter estimates for self-supervised networks trained with the NLR loss. $D_{t}$ and $D_{p}$ are in units of $\mu m^{2}/ms$.


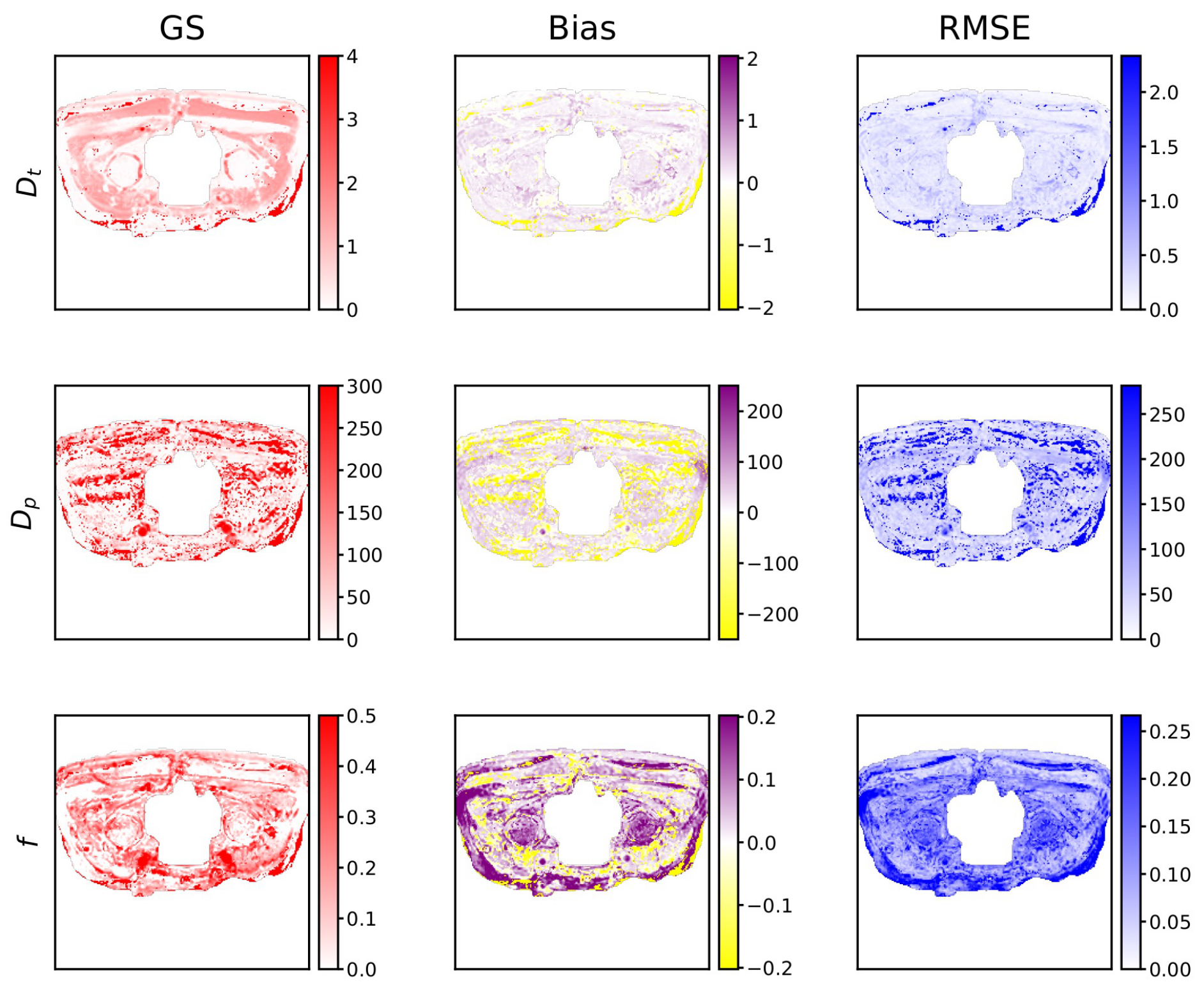


**Fig. S6.** Maps of parameter estimation performance (Bias, RMSE) in high SNR real data with respect to the gold standard (GS) parameter estimates for self-supervised networks trained with the MSE loss. $D_{t}$ and $D_{p}$ are in units of $\mu m^{2}/ms$.

**Fig S7.** Histograms showing the distribution of predicted parameters for networks trained with the NLR loss on low SNR (10) simulated data. Network predictions are shown for 100 noisy data instantiations for three different sets of ground truth parameter values (values indicated at the top of each row). The upper and middle panels show predictions for high and low $D_{t}$ and the lower panel shows predictions for low $D_{p}$. $D_{p}$ errors are highly skewed in all scenarios, leading to a higher bias (mean error) than median error (c.f. Fig. 3, 4 and Fig. S15, S16). $D_{t}$ errors are less skewed and tend to decrease with lower diffusivities (lower $D_{t}$ or lower $D_{p}$).

**Fig. S8.** Comparison of estimation performance in synthetic data at low SNR (10) between self-supervised networks trained with NLR and MSE loss for the IVIM model, in terms of median and inter-quartile range. Points and error bars show the mean and standard deviation of the median or inter-quartile range across unique parameter combinations. NLR points and error bars have been jittered to the right to aid visualisation.

**Fig. S9.** Comparison of estimation performance in synthetic data at high SNR (30) between self-supervised networks trained with NLR and MSE loss for the IVIM model, in terms of median and inter-quartile range. Points and error bars show the mean and standard deviation of the median or inter-quartile range across unique parameter combinations. NLR points and error bars have been jittered to the right to aid visualisation.


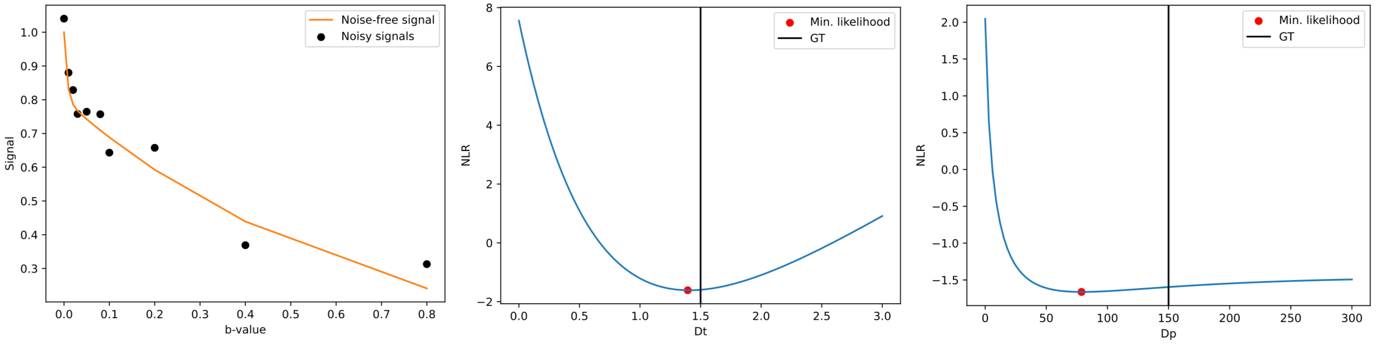


**Fig. S10.** Skewness of the likelihood function for $D_{t}$ and $D_{p}$ parameters. At greater values of $D_{t}$ and $D_{p}$ than the ground truth, the gradient of the likelihood function becomes smaller than at lower values than the ground truth. The signal was generated with ground truth parameters of $D_{t}=1.5 \mu m^{2}/ms$, $D_{p}=150 \mu m^{2}/ms$, $f=0.2$, $S_{0}=1$. Here, the y-axis shows the negative log Rician likelihood (NLR loss).


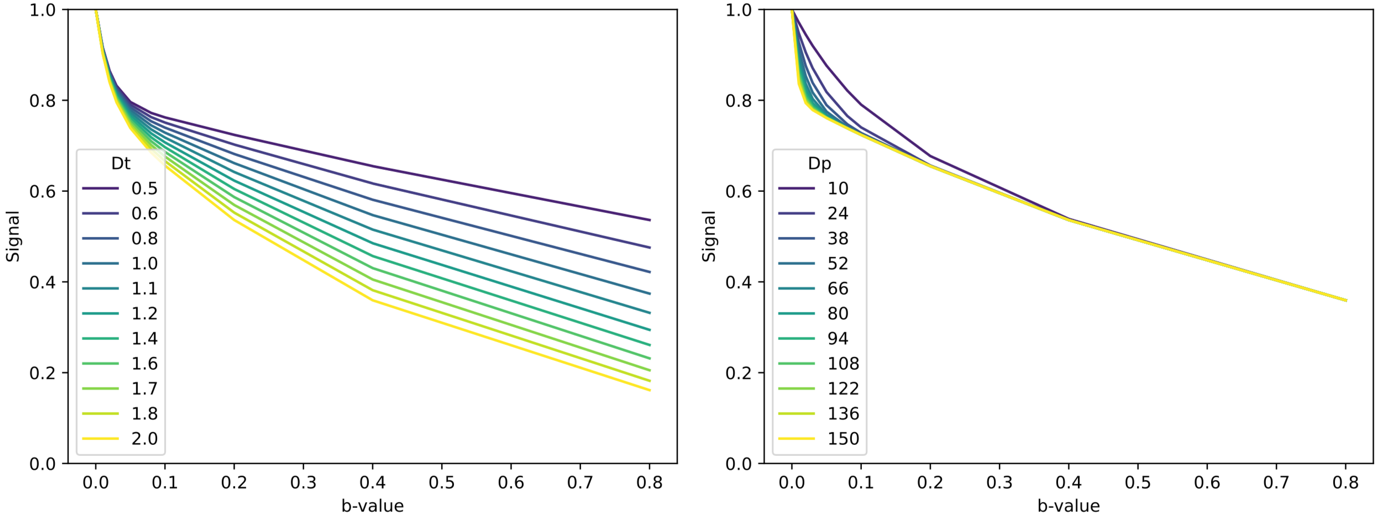


**Fig. S11.** Effect on the signal of varying $D_{t}$ and $D_{p}$. Lines show signal predictions for varying $D_{t}$ (left) and $D_{p}$ (right) values with non-varying ground truth parameters of $D_{t}=1.0 \mu m^{2}/ms$, $D_{p}=50 \mu m^{2}/ms$, $f=0.2$, $S_{0}=1$. The wider range of signals observed for $D_{t}$demonstrates that varying $D_{t}$ has a larger influence on the signal compared to $D_{p}$. b-values are in units of $ms/\mu m^{2}$.

**

**Fig. S12.** Effect of sigma-misestimation on performance of the NLR loss at low SNR (10). Sigma is mis-estimated by a factor of a half to a factor of two. Points and error bars show the mean and standard deviation of the performance metric across unique parameter combinations.

**Fig. S13.** Effect of sigma-misestimation on performance of the NLR loss at high SNR (30). Sigma is mis-estimated by a factor of a half to a factor of two. Points and error bars show the mean and standard deviation of the performance metric across unique parameter combinations.


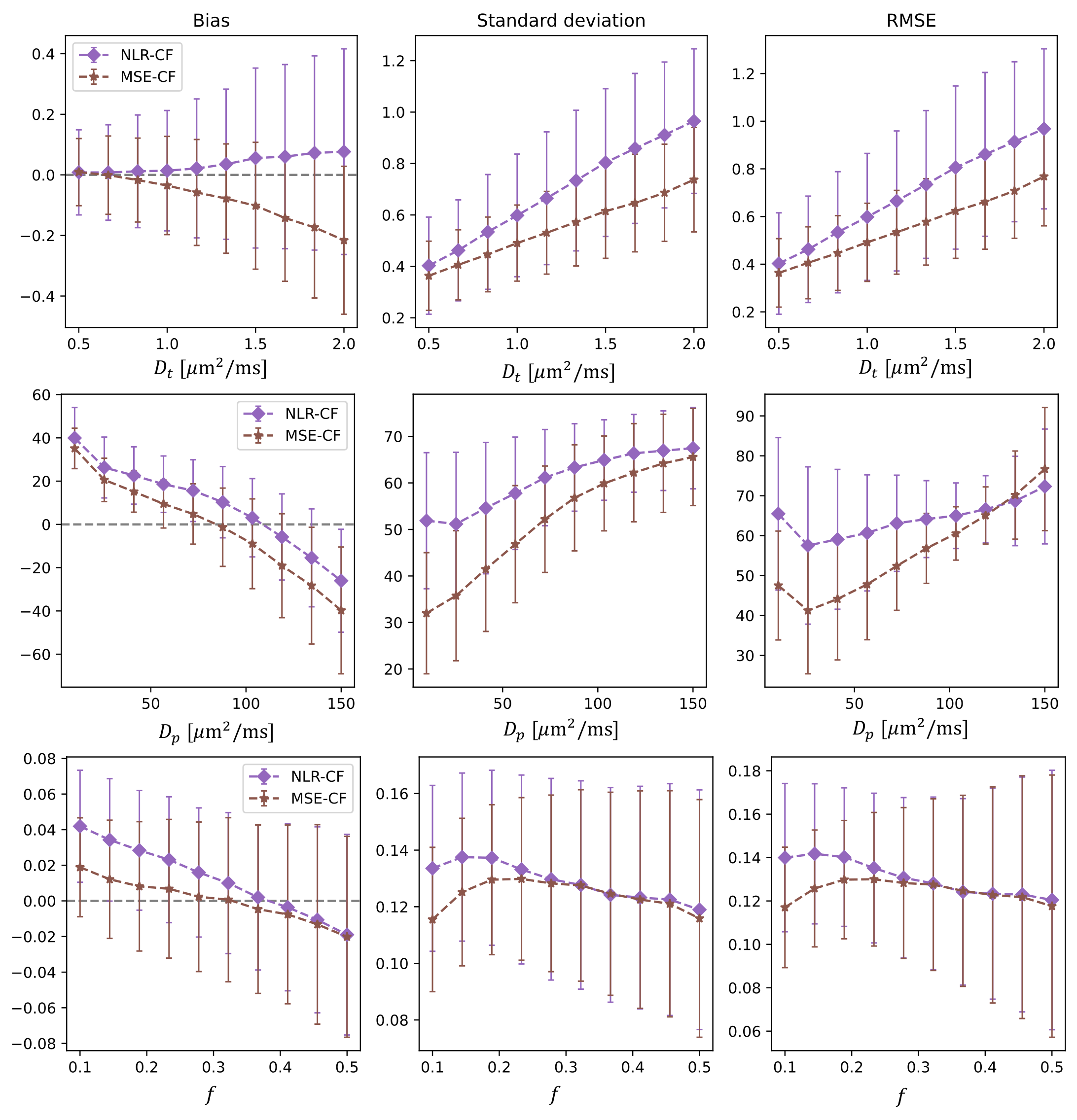


**Fig. S14.** Comparison of estimation performance in synthetic low SNR (10) data between conventional voxel-wise fitting with NLR and MSE loss for the IVIM model. Points and error bars show the mean and standard deviation of the performance metric across unique parameter combinations.


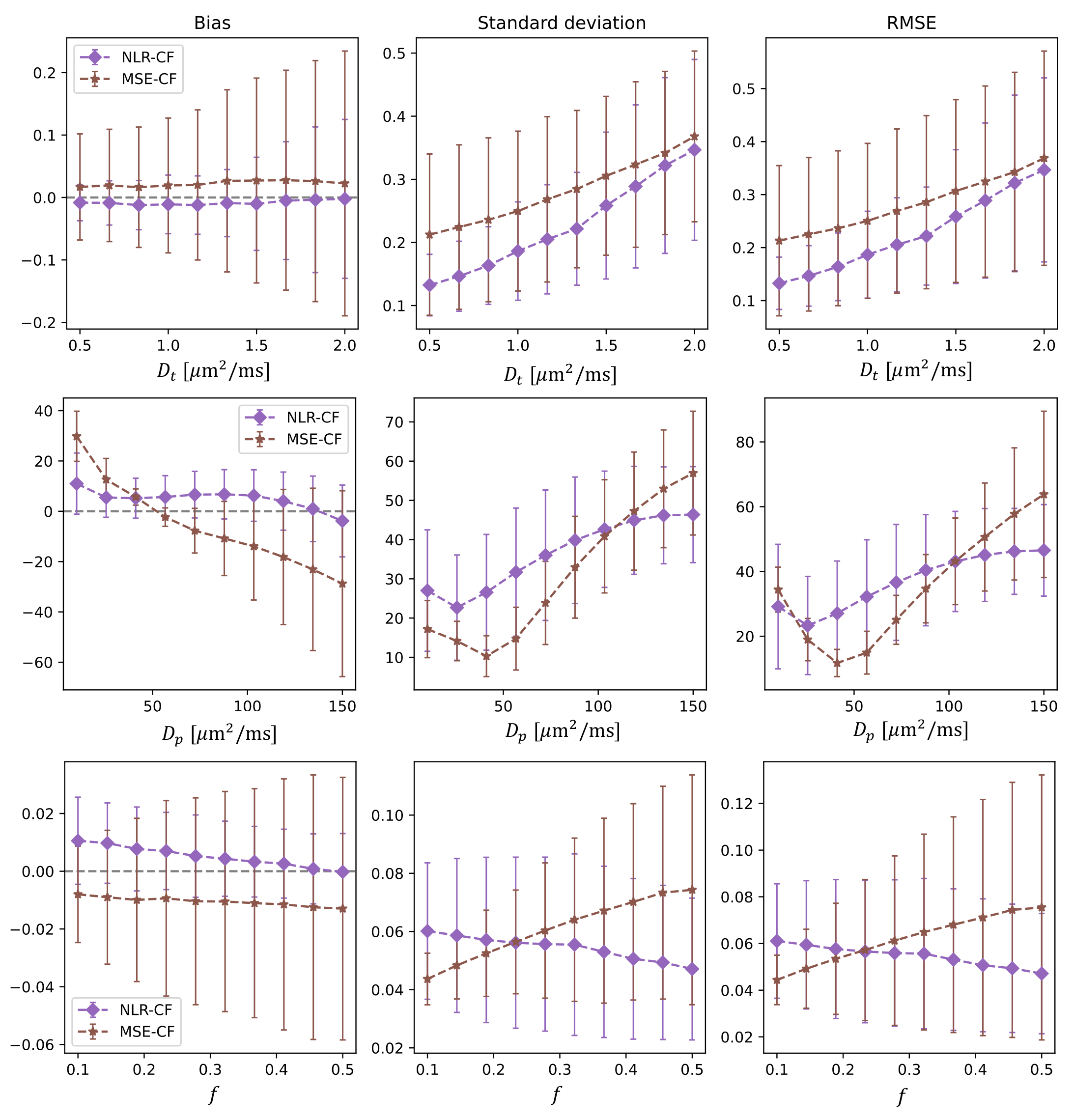


**Fig. S15.** Comparison of estimation performance in synthetic high SNR (30) data between conventional voxel-wise fitting with NLR and MSE loss for the IVIM model. Points and error bars show the mean and standard deviation of the performance metric across unique parameter combinations.

**Fig. S16.** Boxplots of fitting errors in parameter estimates from conventional voxel-wise fitting for synthetic data at SNRs of 30, 20, 10, 7.5 and 5 for the IVIM model. The line shows the median error across all estimates and the box shows the inter-quartile range. Whiskers extend to the most extreme data point within 1.5 times the inter-quartile range from the median.
