# Supplementary figures and images for: Rician Likelihood Loss for Quantitative MRI With Self‐Supervised Deep Learning
Source: NMR Biomed. 2025 Sep 3;38(10):e70136. doi: 10.1002/nbm.70136 (PMC12421220; doi:10.1002/nbm.70136)

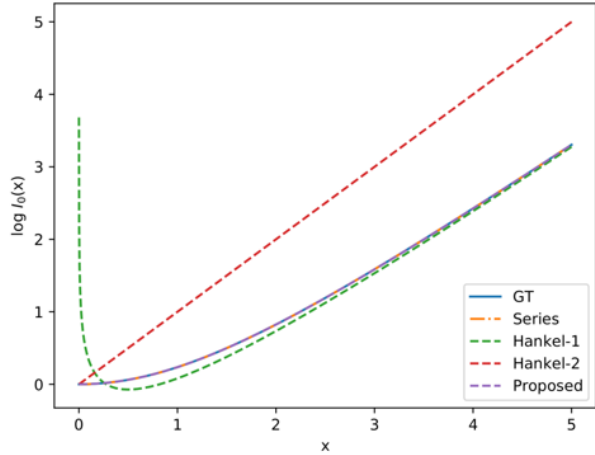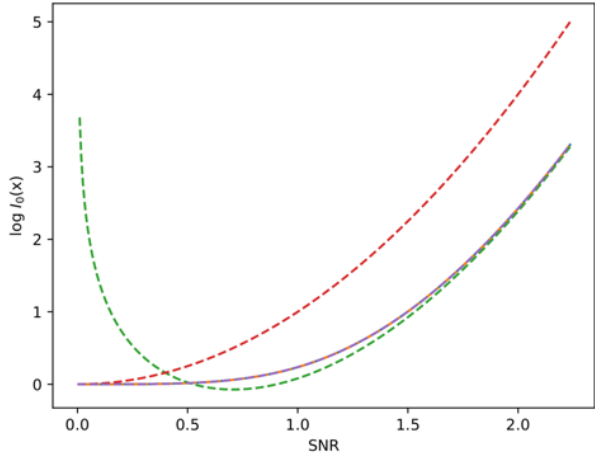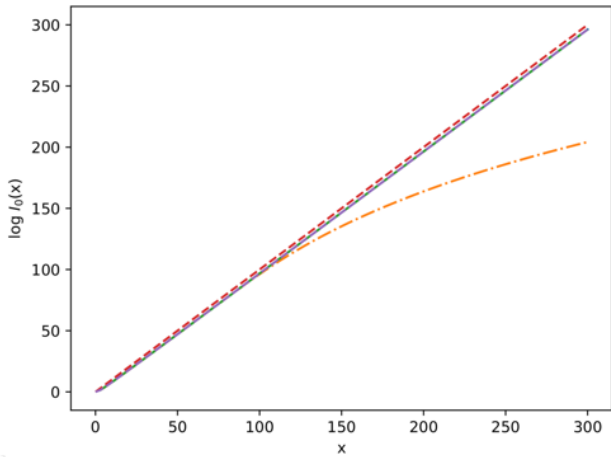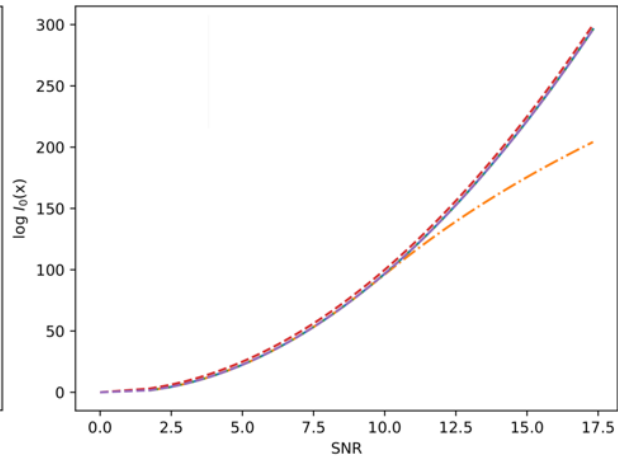

Supplement: Supplementary file 3 — Figure S1: logI0x approximations, as a function of input, x, and SNR ≈x. In the left column, plots are a function of x. In the right column, plots are a function of SNR. The top and bottom rows show different ranges of SNRs—low and high, respectively. The ground truth (GT, blue line) was computed using 1000 summations of the series expansion, which converges in value for the range of x shown. Note the GT line is only partially visible as it overlaps with the approximations. In the bottom left plot (high SNR as a function of x), it is fully obscured by the Proposed and Hankel‐1 lines. [file NBM-38-e70136-s011.pdf]

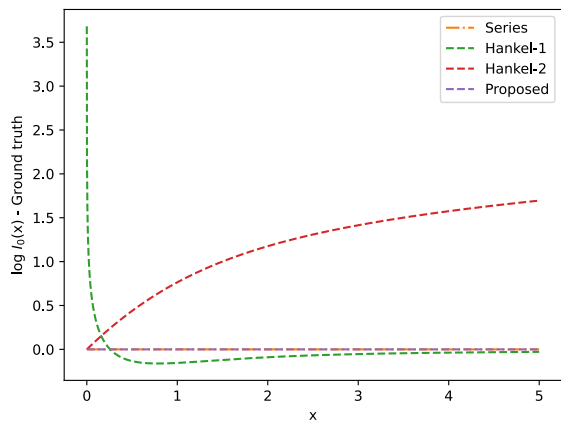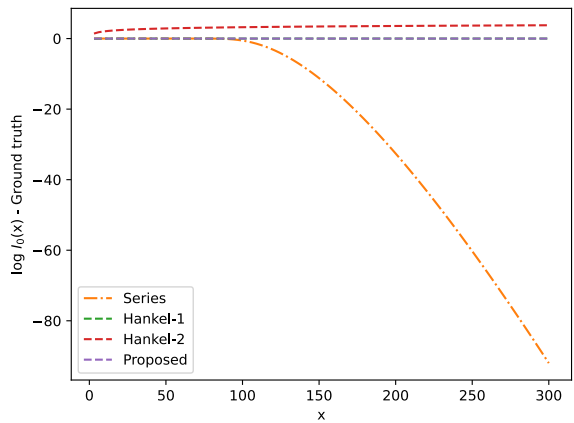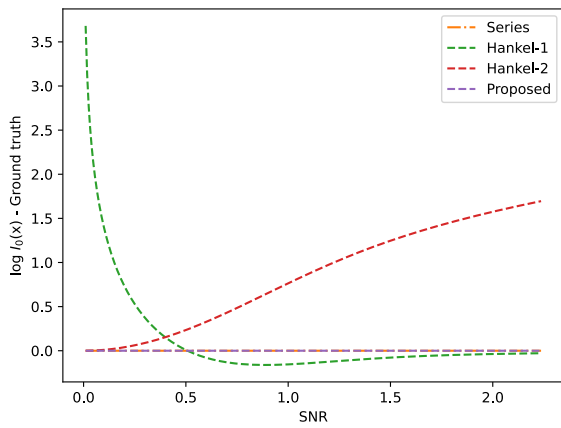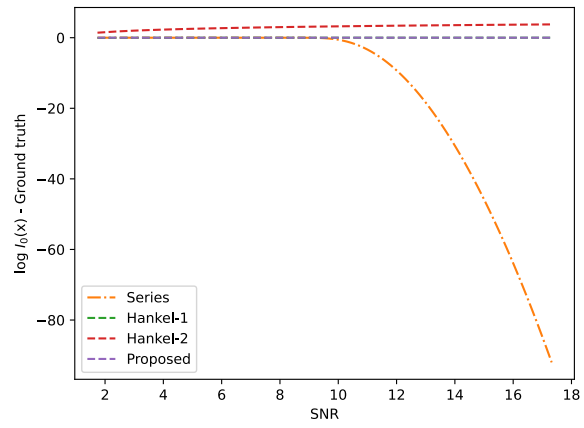

Supplement: Supplementary file 4 — Figure S2: logI0x approximations minus ground truth, as a function of input, x, and SNR ≈x. In the left column, plots are a function of x. In the right column, plots are a function of SNR. The top and bottom rows show different ranges of SNRs—low and high, respectively. The ground truth was computed using 1000 summations of the series expansion, which converges in value for the range of x shown. [file NBM-38-e70136-s017.pdf]

# Not initialised

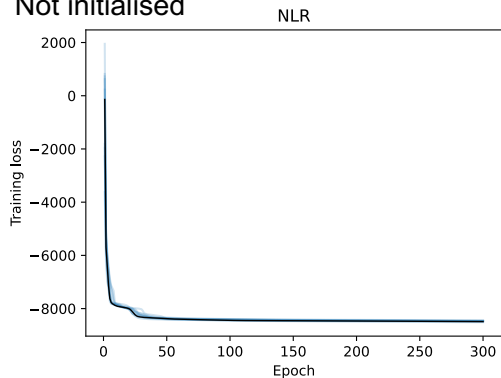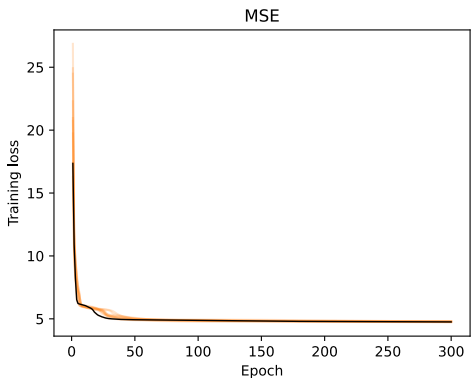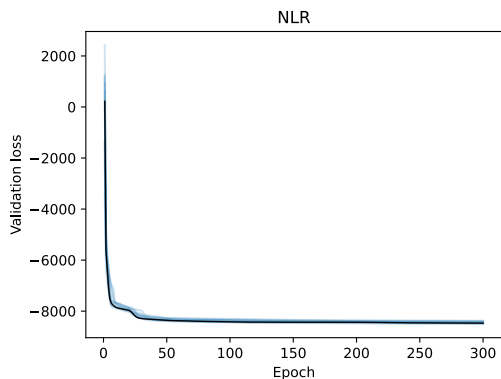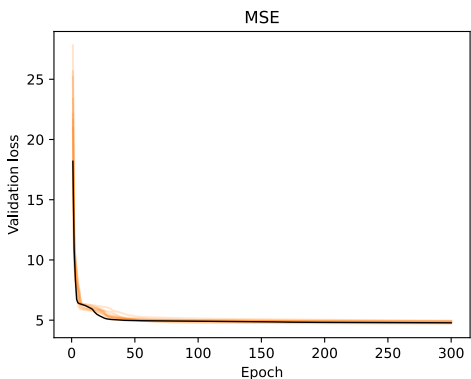

# Initialised

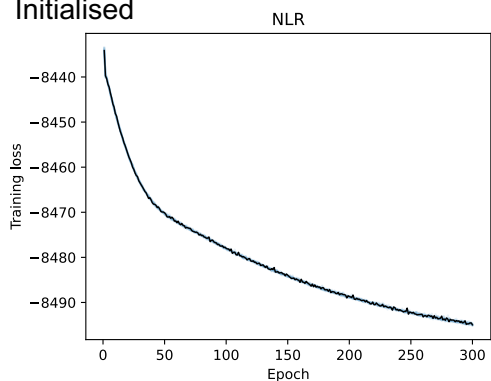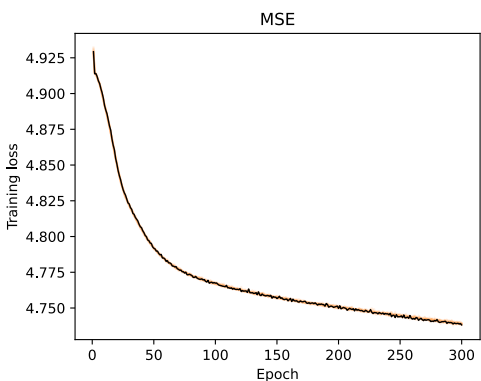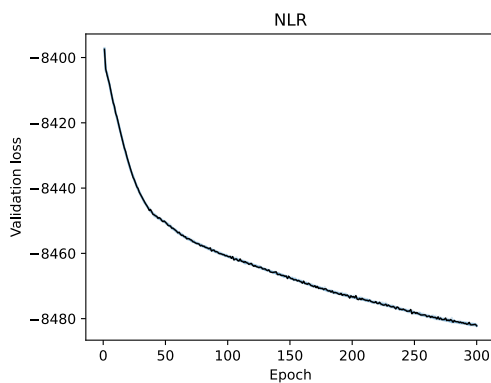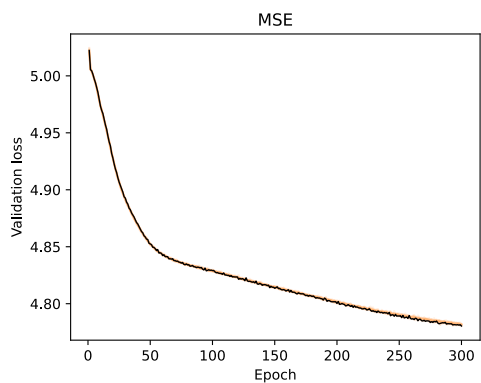

Supplement: Supplementary file 5 — Figure S3: Training curves for the NLR and MSE loss on low SNR data. The upper panel shows loss curves with no initialisation, and the lower panel shows loss curves with initialisation. [file NBM-38-e70136-s015.pdf]

GS

 $D_t$ 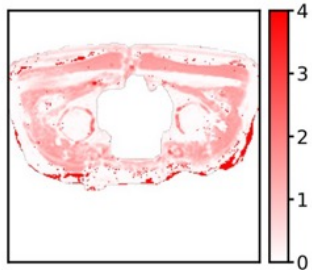

Bias

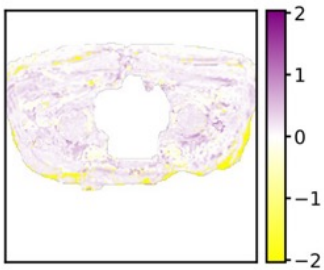

RMSE

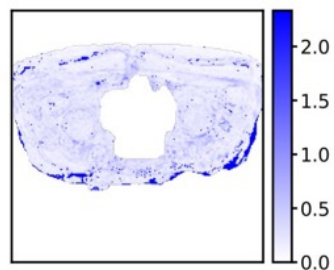 $D_p$ 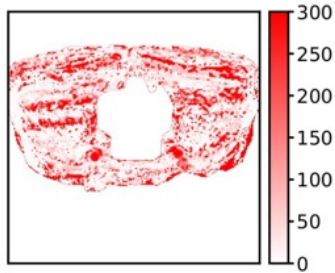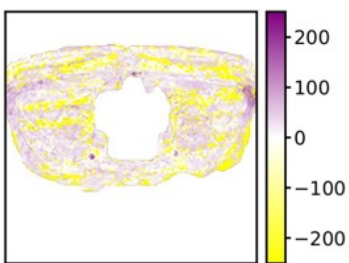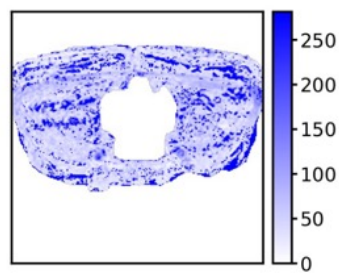 $f$ 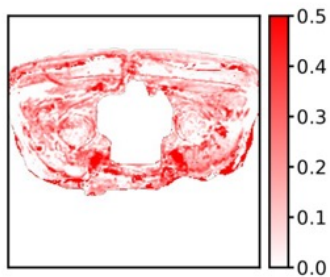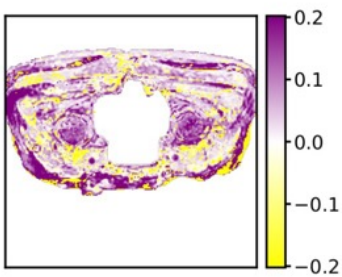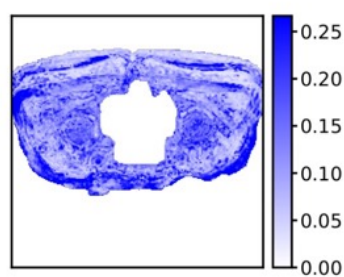

Supplement: Supplementary file 7 — Figure S5: Maps of parameter estimation performance (Bias, RMSE) in high SNR real data with respect to the gold standard (GS) parameter estimates for self‐supervised networks trained with the NLR loss. Dt and Dp are in units of μm2/ms. [file NBM-38-e70136-s007.pdf]

GS

Bias

RMSE

 $D_t$ 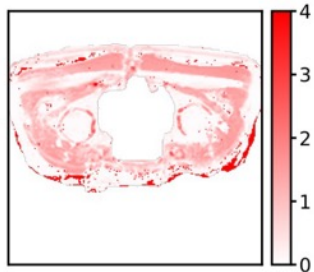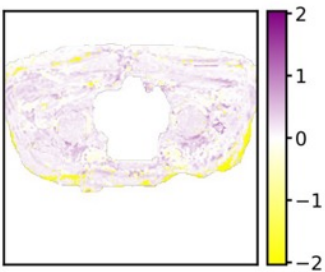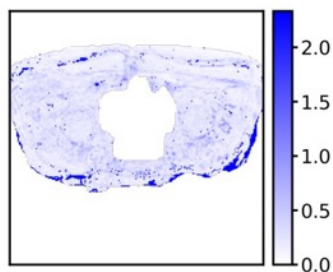 $D_p$ 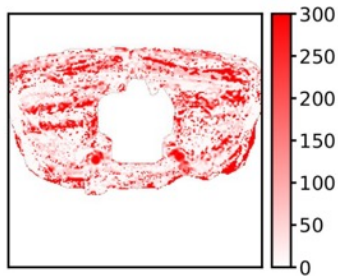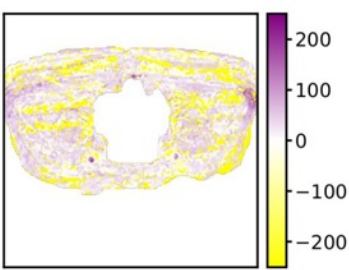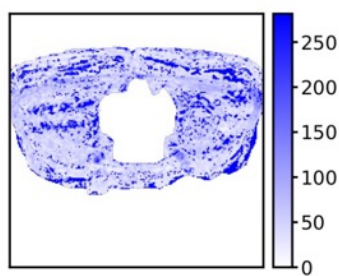 $f$ 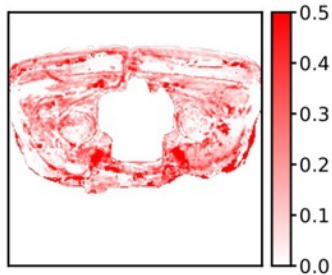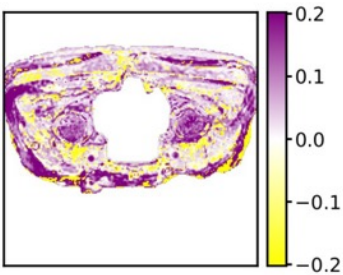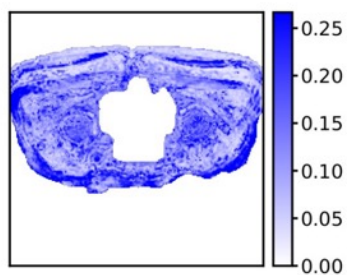

Supplement: Supplementary file 8 — Figure S6: Maps of parameter estimation performance (Bias, RMSE) in high SNR real data with respect to the gold standard (GS) parameter estimates for self‐supervised networks trained with the MSE loss. Dt and Dp are in units of μm2/ms. [file NBM-38-e70136-s003.pdf]

Median Error

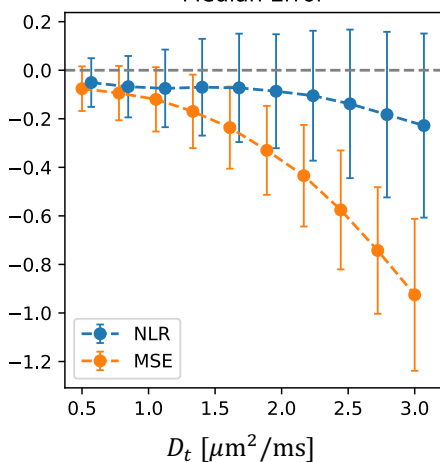

Inter-Quartile Range

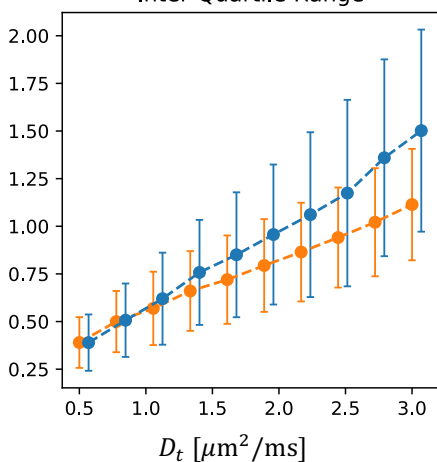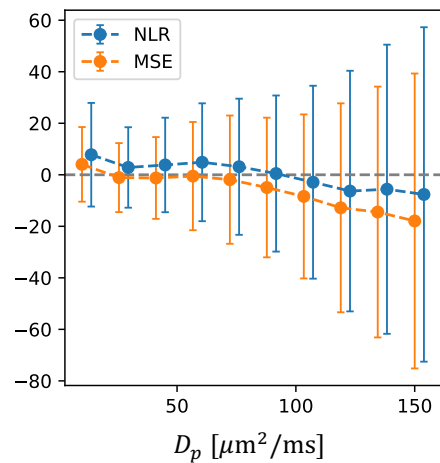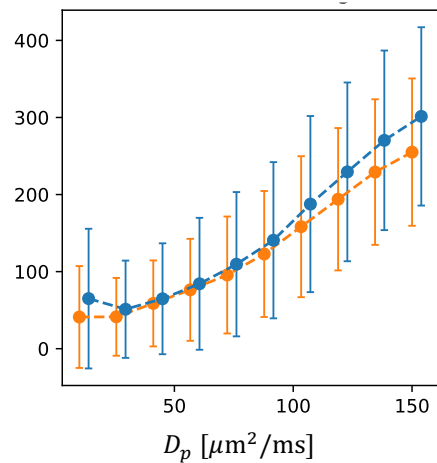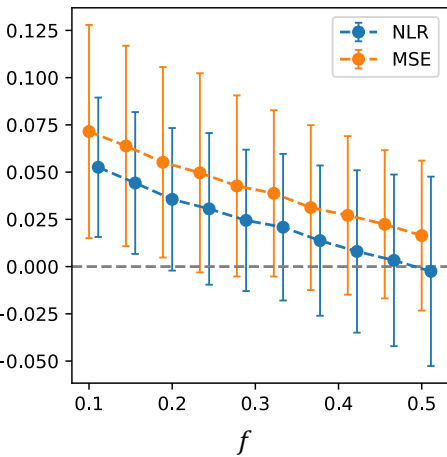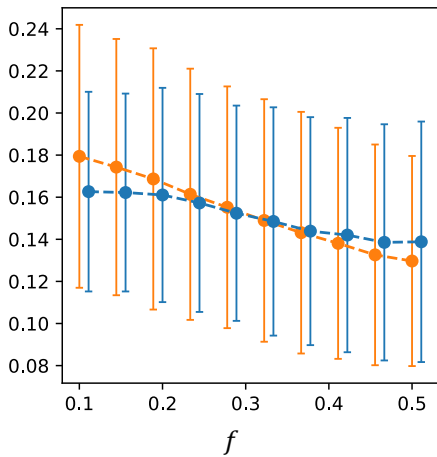

Supplement: Supplementary file 10 — Figure S8: Comparison of estimation performance in synthetic data at low SNR (10) between self‐supervised networks trained with NLR and MSE loss for the IVIM model, in terms of median and interquartile range. Points and error bars show the mean and standard deviation of the median or interquartile range across unique parameter combinations. NLR points and error bars have been jittered to the right to aid visualisation. [file NBM-38-e70136-s014.pdf]

Median Error

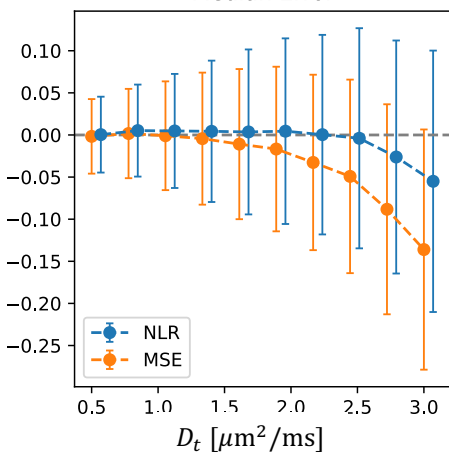

Inter-Quartile Range

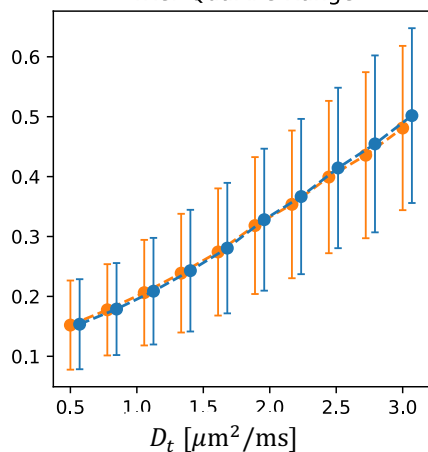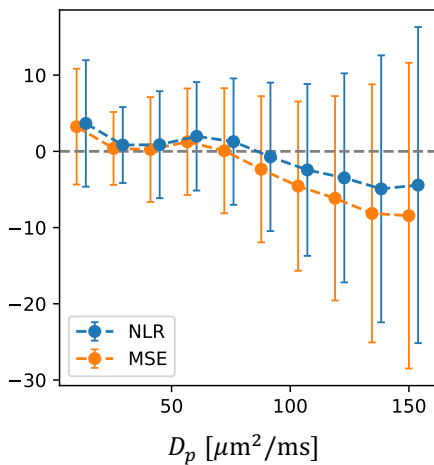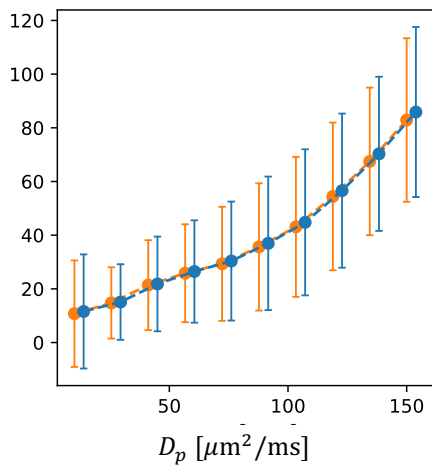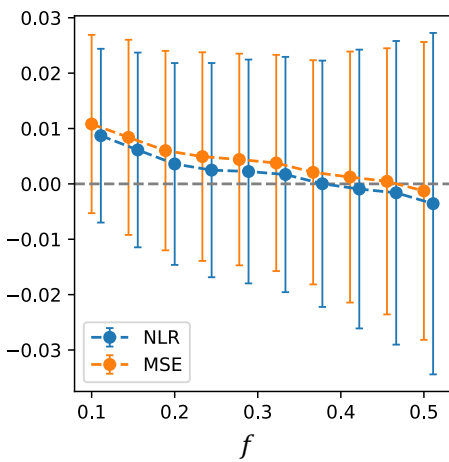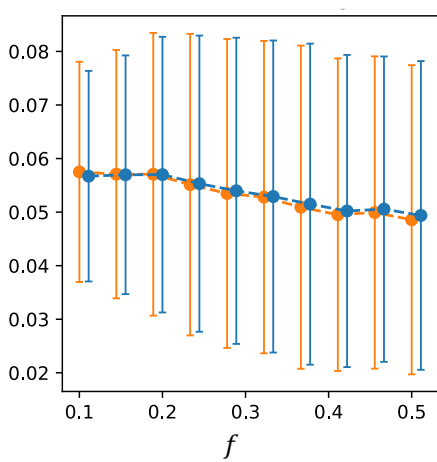

Supplement: Supplementary file 11 — Figure S9: Comparison of estimation performance in synthetic data at high SNR (30) between self‐supervised networks trained with NLR and MSE loss for the IVIM model, in terms of median and interquartile range. Points and error bars show the mean and standard deviation of the median or interquartile range across unique parameter combinations. NLR points and error bars have been jittered to the right to aid visualisation. [file NBM-38-e70136-s001.pdf]

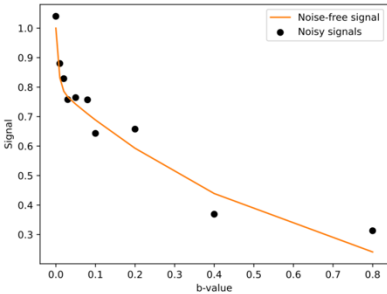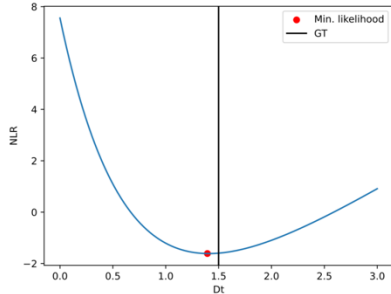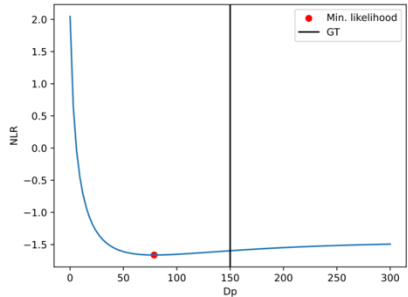

Supplement: Supplementary file 12 — Figure S10: Skewness of the likelihood function for Dt and Dp parameters. At greater values of Dt and Dp than the ground truth, the gradient of the likelihood function becomes smaller than at lower values than the ground truth. The signal was generated with ground truth parameters of Dt=1.5μm2/ms, Dp=150μm2/ms, f=0.2, S0=1. Here, the y‐axis shows the negative log Rician likelihood (NLR loss). [file NBM-38-e70136-s006.pdf]

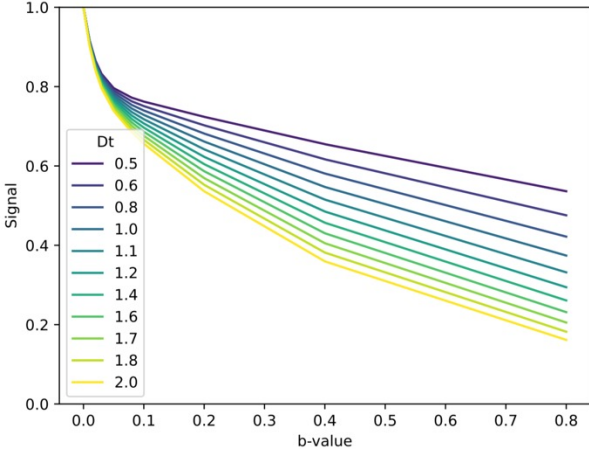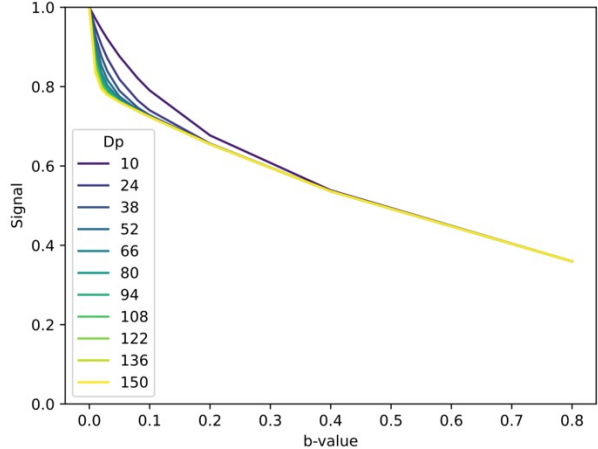

Supplement: Supplementary file 13 — Figure S11: Effect on the signal of varying Dt and Dp. Lines show signal predictions for varying Dt (left) and Dp (right) values with nonvarying ground truth parameters of Dt=1.0μm2/ms, Dp=50μm2/ms, f=0.2, S0=1. The wider range of signals observed for Dt demonstrates that varying Dt has a larger influence on the signal compared with Dp. b‐values are in units of ms/μm2. [file NBM-38-e70136-s002.pdf]

Bias

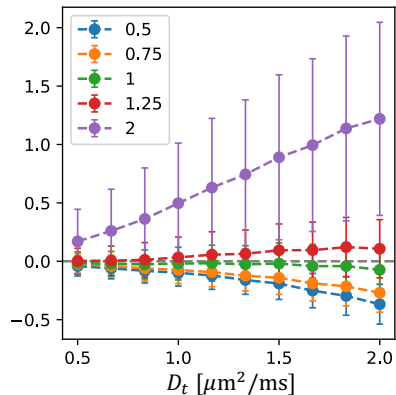

Standard deviation

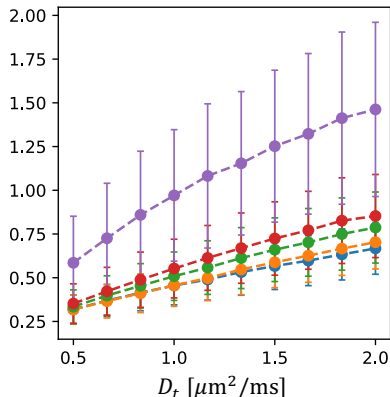

RMSE

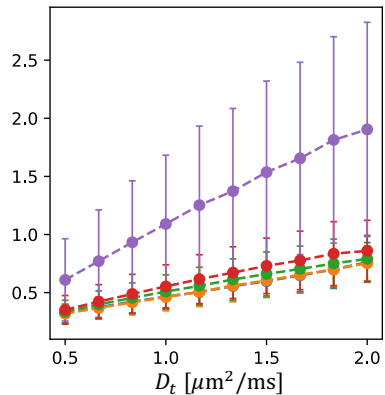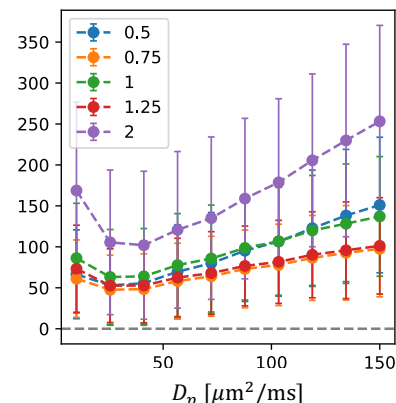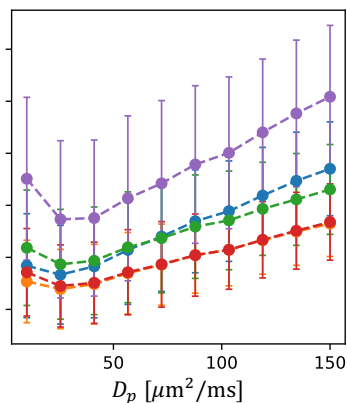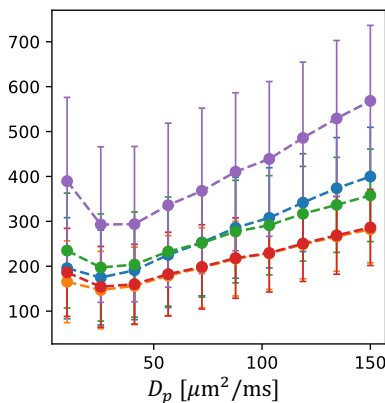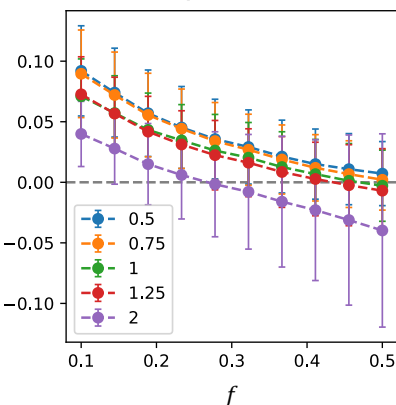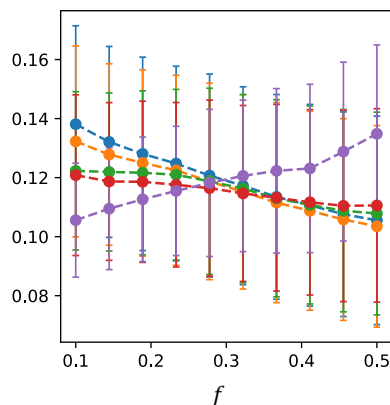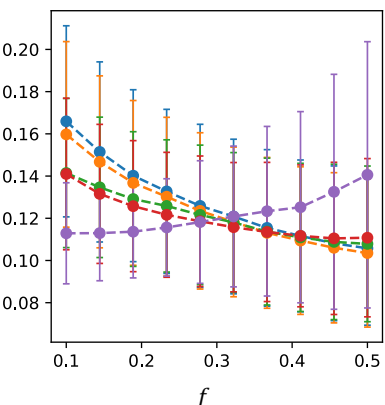

Supplement: Supplementary file 14 — Figure S12: Effect of sigma‐misestimation on performance of the NLR loss at low SNR (10). Sigma is misestimated by a factor of a half to a factor of two. Points and error bars show the mean and standard deviation of the performance metric across unique parameter combinations. [file NBM-38-e70136-s018.pdf]

Bias

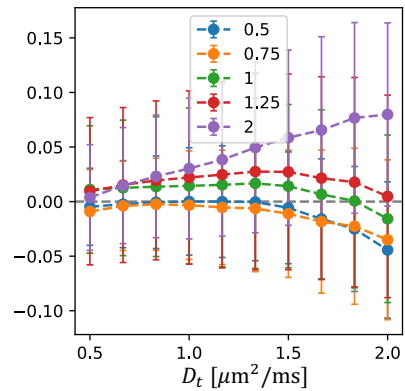

Standard deviation

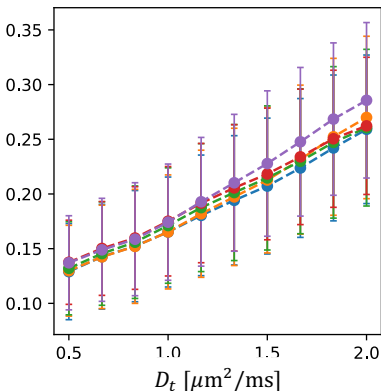

RMSE

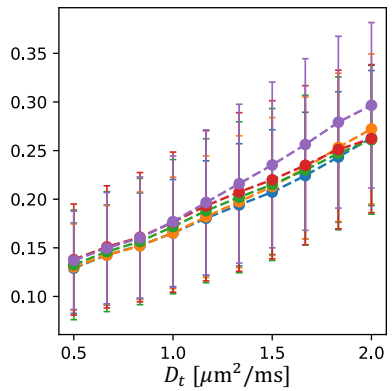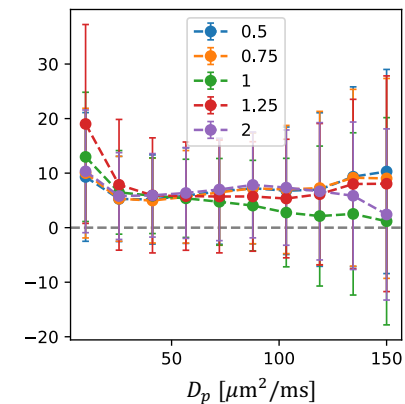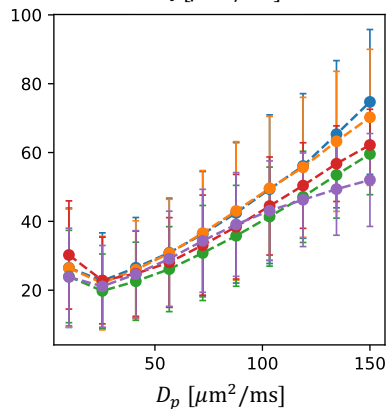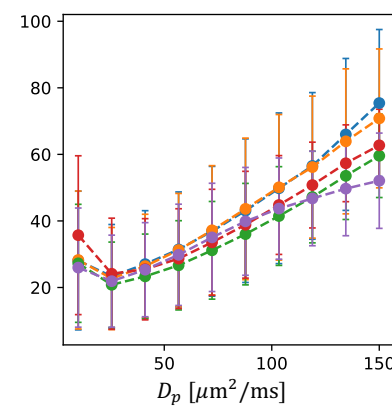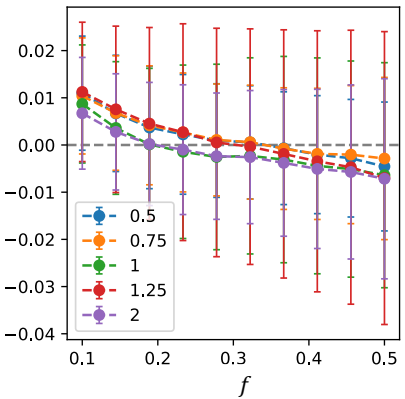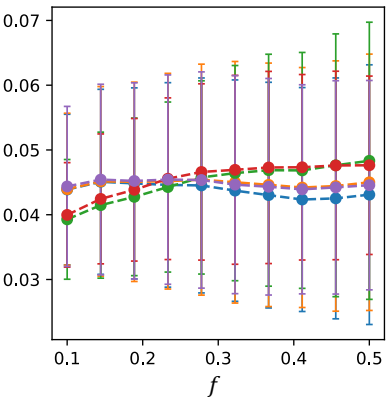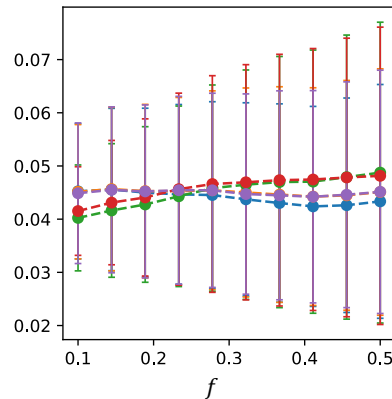

Supplement: Supplementary file 15 — Figure S13: Effect of sigma‐misestimation on performance of the NLR loss at high SNR (30). Sigma is misestimated by a factor of a half to a factor of two. Points and error bars show the mean and standard deviation of the performance metric across unique parameter combinations. [file NBM-38-e70136-s009.pdf]

Bias

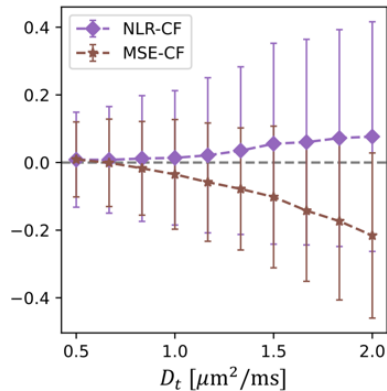

Standard deviation

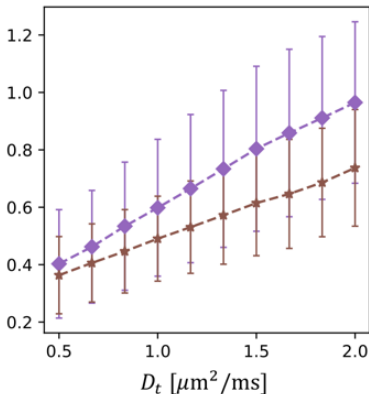

RMSE

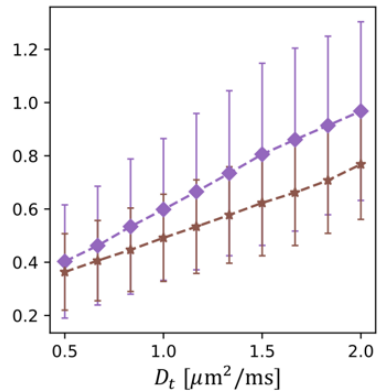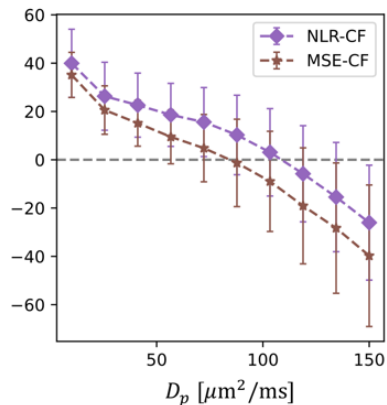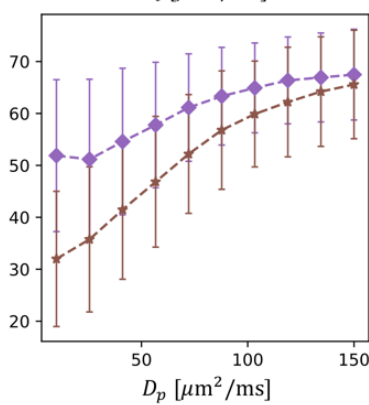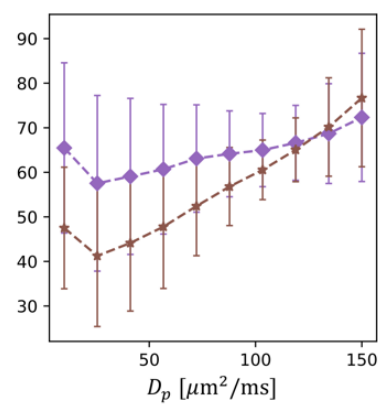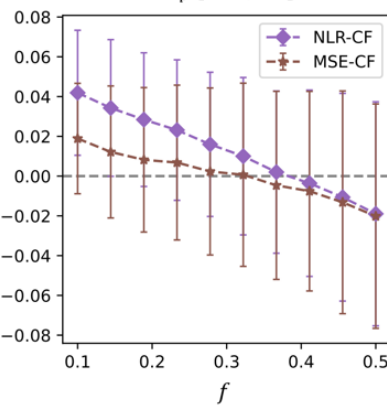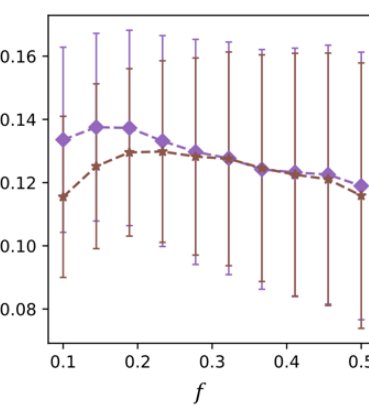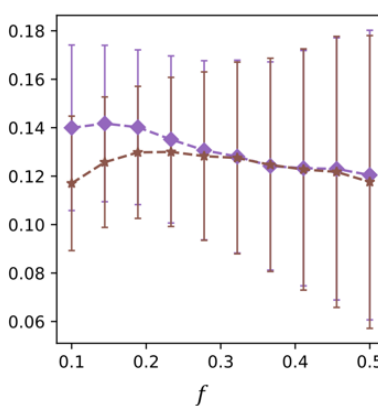

Supplement: Supplementary file 16 — Figure S14: Comparison of estimation performance in synthetic low SNR (10) data between conventional voxel‐wise fitting with NLR and MSE loss for the IVIM model. Points and error bars show the mean and standard deviation of the performance metric across unique parameter combinations. [file NBM-38-e70136-s016.pdf]

Bias

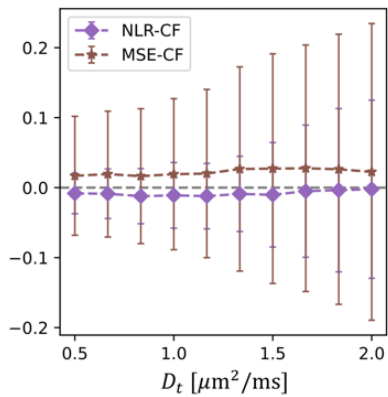

Standard deviation

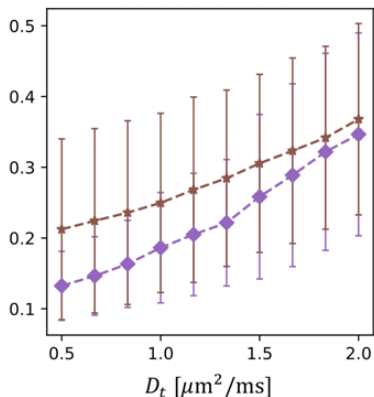

RMSE

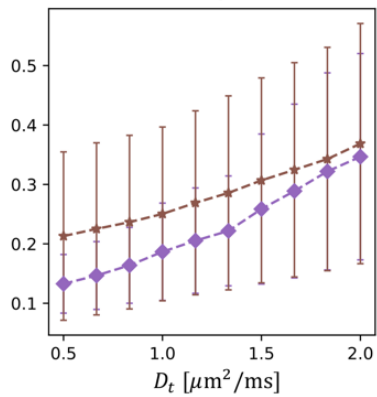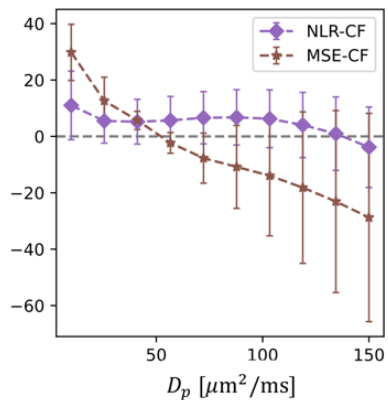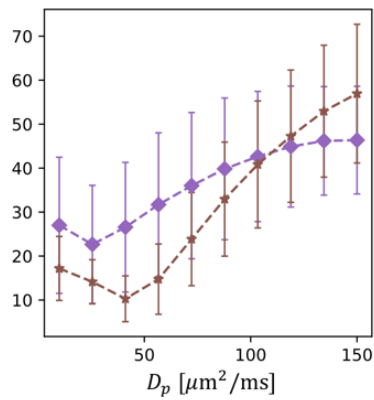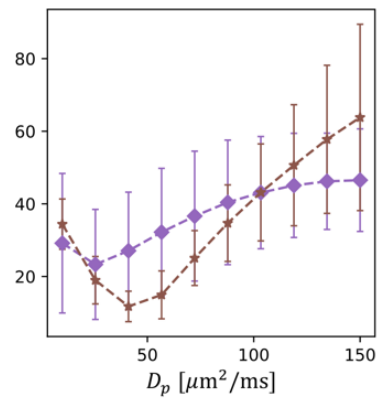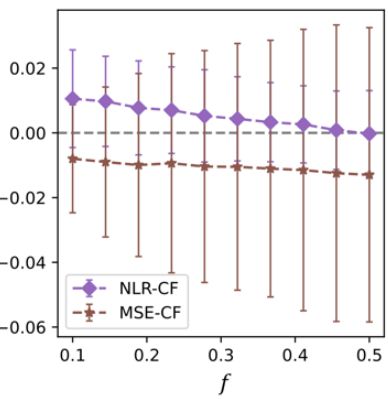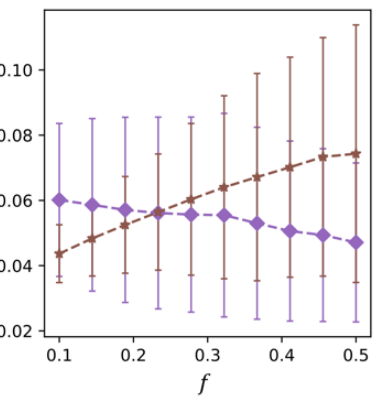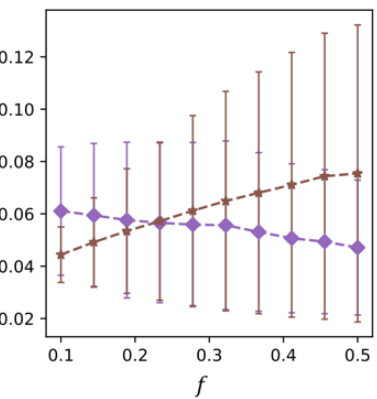

Supplement: Supplementary file 17 — Figure S15: Comparison of estimation performance in synthetic high SNR (30) data between conventional voxel‐wise fitting with NLR and MSE loss for the IVIM model. Points and error bars show the mean and standard deviation of the performance metric across unique parameter combinations. [file NBM-38-e70136-s012.pdf]

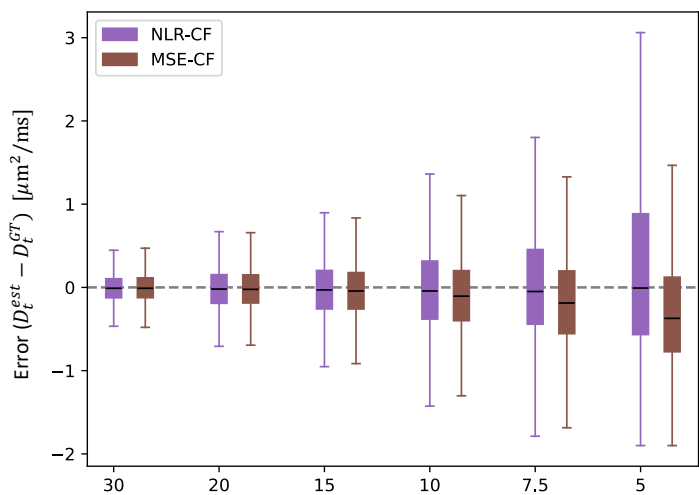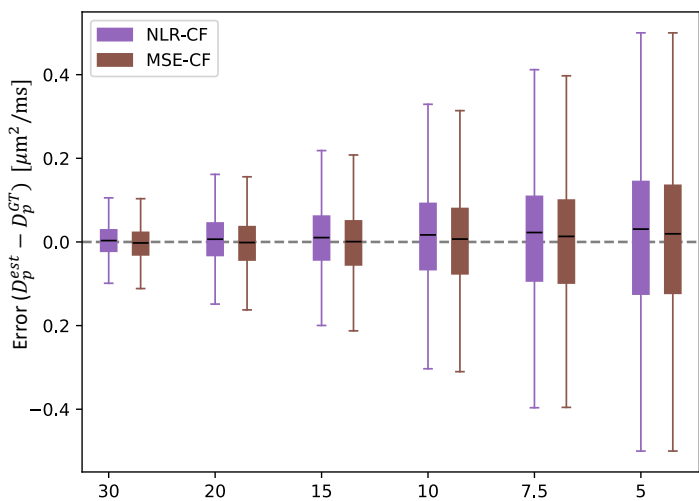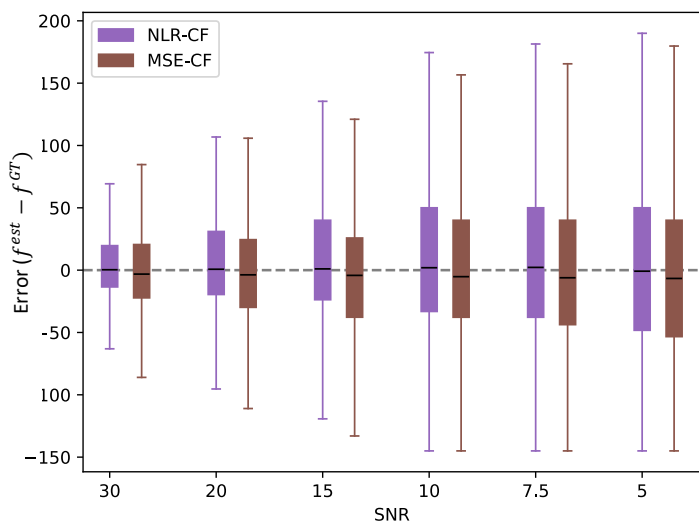

Supplement: Supplementary file 18 — Figure S16: Boxplots of fitting errors in parameter estimates from conventional voxel‐wise fitting for synthetic data at SNRs of 30, 20, 10, 7.5 and 5 for the IVIM model. The line shows the median error across all estimates and the box shows the interquartile range. Whiskers extend to the most extreme data point within 1.5 times the interquartile range from the median. [file NBM-38-e70136-s010.pdf]
