# Supplementary material for: Rician Likelihood Loss for Quantitative MRI With Self‐Supervised Deep Learning
Source: NMR Biomed. 2025 Sep 3;38(10):e70136. doi: 10.1002/nbm.70136 (PMC12421220; doi:10.1002/nbm.70136)
Supplement: Supplementary file 6 — Figure S4: Comparison of estimation performance in high SNR real data with respect to the gold standard maps between self‐supervised networks trained with NLR and MSE loss for the IVIM model. Points and error bars show the mean and standard deviation of the performance metric across binned parameter values. MSE points and error bars have been jittered to the right to aid visualisation. [file NBM-38-e70136-s008.pdf]

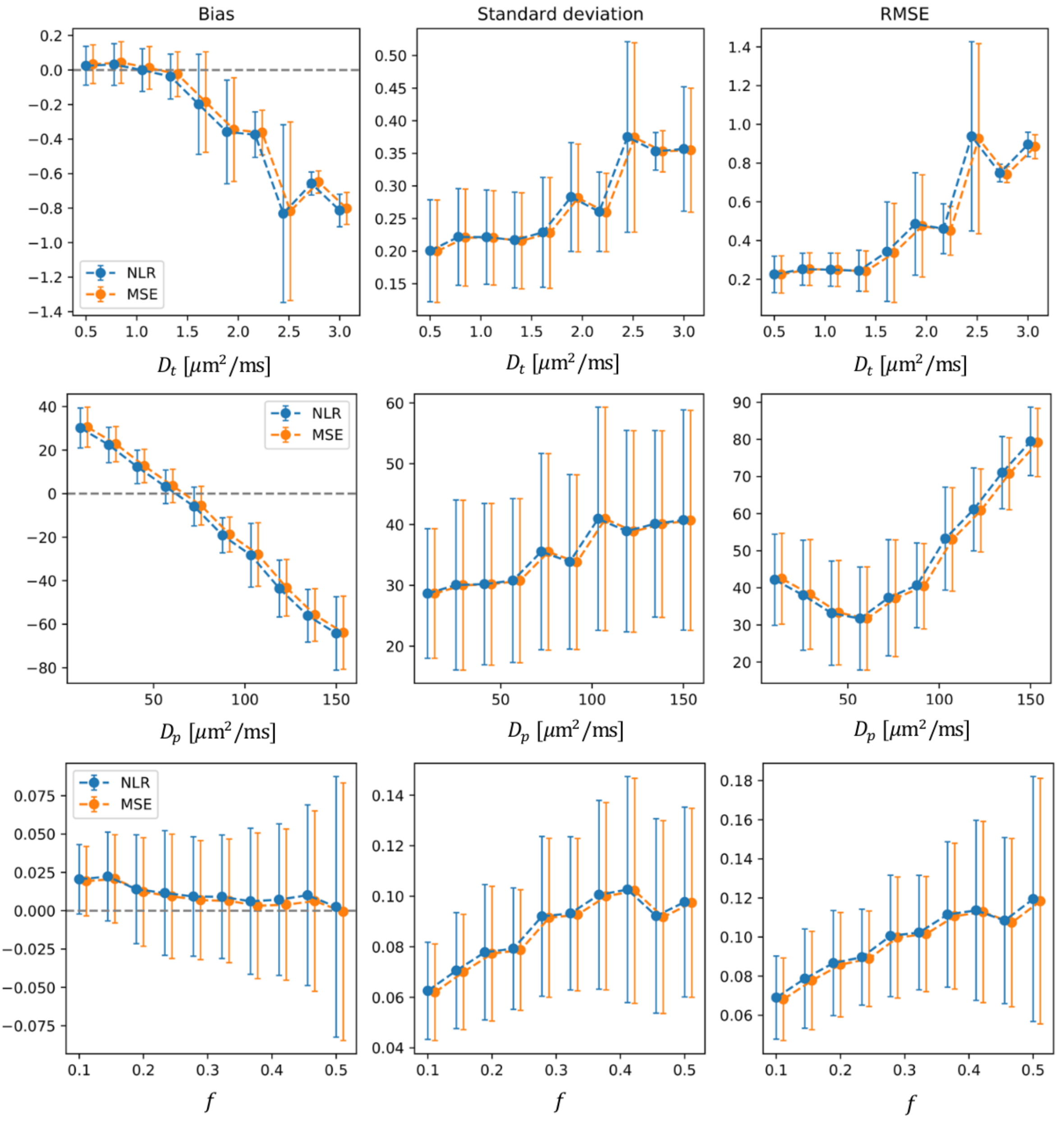
Bias
Standard deviation
RMSE
NLR
MSE
$D_t$ [$\mu$m$^2$/ms]
$D_p$ [$\mu$m$^2$/ms]
$f$
