# Supplementary material for: Rician Likelihood Loss for Quantitative MRI With Self‐Supervised Deep Learning
Source: NMR Biomed. 2025 Sep 3;38(10):e70136. doi: 10.1002/nbm.70136 (PMC12421220; doi:10.1002/nbm.70136)

## $D_t = 2.5\ \mu m^2/mm$, $D_t = 75\ \mu m^2/mm$, $f = 0.3$, $S_0 = 1$

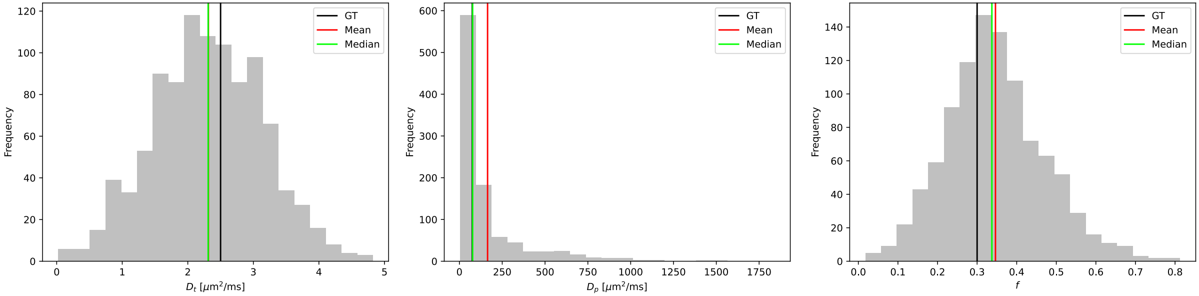


## $D_t = 0.5\ \mu m^2/mm$, $D_t = 75\ \mu m^2/mm$, $f = 0.3$, $S_0 = 1$

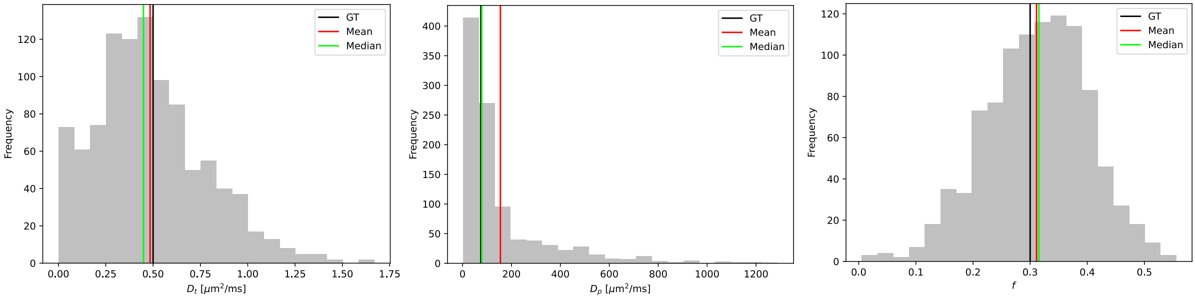


## $D_t = 1.5\ \mu m^2/mm$, $D_t = 20\ \mu m^2/mm$, $f = 0.3$, $S_0 = 1$

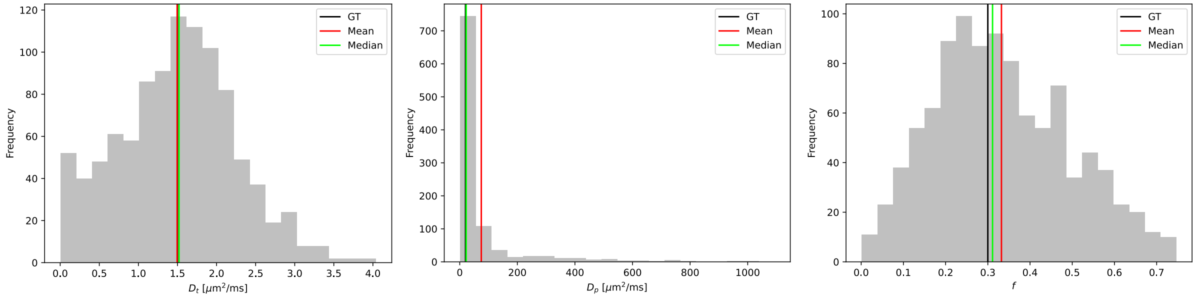

Supplement: Supplementary file 9 — Figure S7: Histograms showing the distribution of predicted parameters for networks trained with the NLR loss on low SNR (10) simulated data. Network predictions are shown for 100 noisy data instantiations for three different sets of ground truth parameter values (values indicated at the top of each row). The upper and middle panels show predictions for high and low Dt, and the lower panel shows predictions for low Dp. Dp errors are highly skewed in all scenarios, leading to a higher bias (mean error) than median error (cf. Figures 3, 4 S15 and S16). Dt errors are less skewed and tend to decrease with lower diffusivities (lower Dt or lower Dp). [file NBM-38-e70136-s005.pdf]
